# Phloem unloading via the apoplastic pathway is essential for shoot distribution of root-synthesized cytokinins

**DOI:** 10.1093/plphys/kiab188

**Published:** 2021-04-27

**Authors:** Jiangzhe Zhao, Bingli Ding, Engao Zhu, Xiaojuan Deng, Mengyuan Zhang, Penghong Zhang, Lu Wang, Yangshuo Dai, Shi Xiao, Cankui Zhang, Chang-Jun Liu, Kewei Zhang

**Affiliations:** 1 Institute of Plant Genetics and Developmental Biology, Zhejiang Provincial Key Laboratory of Biotechnology on Specialty Economic Plants, College of Chemistry and Life Sciences, Zhejiang Normal University, Jinhua, Zhejiang 321004, P.R. China; 2 State Key Laboratory of Biocontrol, Guangdong Provincial Key Laboratory of Plant Resources, Collaborative Innovation Center of Genetics and Development, School of Life Sciences, Sun Yat-sen University, Guangzhou 510275, P.R. China; 3 Department of Agronomy and Purdue Center for Plant Biology, Purdue University, West Lafayette, Indiana, USA; 4 Biology Department, Brookhaven National Laboratory, Upton, New York 11973, USA

## Abstract

Root-synthesized cytokinins are transported to the shoot and regulate the growth, development, and stress responses of aerial tissues. Previous studies have demonstrated that Arabidopsis (*Arabidopsis thaliana*) ATP binding cassette (ABC) transporter G family member 14 (AtABCG14) participates in xylem loading of root-synthesized cytokinins. However, the mechanism by which these root-derived cytokinins are distributed in the shoot remains unclear. Here, we revealed that AtABCG14-mediated phloem unloading through the apoplastic pathway is required for the appropriate shoot distribution of root-synthesized cytokinins in Arabidopsis. Wild-type rootstocks grafted to *atabcg14* scions successfully restored trans-zeatin xylem loading. However, only low levels of root-synthesized cytokinins and induced shoot signaling were rescued. Reciprocal grafting and tissue-specific genetic complementation demonstrated that AtABCG14 disruption in the shoot considerably increased the retention of root-synthesized cytokinins in the phloem and substantially impaired their distribution in the leaf apoplast. The translocation of root-synthesized cytokinins from the xylem to the phloem and the subsequent unloading from the phloem is required for the shoot distribution and long-distance shootward transport of root-synthesized cytokinins. This study revealed a mechanism by which the phloem regulates systemic signaling of xylem-mediated transport of root-synthesized cytokinins from the root to the shoot.

## Teaser

Phloem unloading via the apoplastic pathway is essential for shoot distribution and long-distance translocation of root-synthesized cytokinins from the root to the shoot through the xylem.

## Introduction

Phytohormones such as cytokinins are small signaling molecules produced by various essential metabolic pathways that enable sessile plants to grow and adapt to highly variable environments (Robert and Friml, 2009; Santner et al., 2009). Cytokinins are *N^6^*-substituted adenine derivatives that are essential for plant growth, development, and stress response (Sakakibara, 2006; Hwang et al., 2012). The most common cytokinin derivatives in plants are *N^6^*-(Δ^2^-isopentenyl) adenine (iP), trans-zeatin (tZ), cis-zeatin (cZ), and dihydrozeatin, which differ in the end of their prenyl side chains (Sakakibara, 2006; Kiba et al., 2013). In Arabidopsis, the major active types of cytokinins are iP and tZ (Sakakibara, 2006; Osugi et al., 2017), and their respective precursors are iP-riboside (iPR) and tZ-riboside (tZR), which may be systemically transported or serve as storage forms (Hirose et al., 2008). Grafting experiments and functional analyses of cytokinin biosynthesis genes suggested that the tZ-type cytokinins are mainly synthesized in the roots, whereas the iP-type cytokinins are synthesized in both the shoots and the roots of Arabidopsis (Matsumoto-Kitano et al., 2008; Kiba et al., 2013).

De novo cytokinin biosynthesis occurs only in certain cell types (Kang et al., 2017). Newly synthesized cytokinins must be delivered from the source to sink cells for signaling via diffusion and/or active translocation (De Rybel et al., 2014; Kang et al., 2017; Liu et al., 2019). The long-distance transport process complemented by several short-range apoplastic, symplastic, and transcellular transport mechanisms is the main phytohormone transport pathway (Robert and Friml, 2009). The main long-distance transport streams include root-to-shoot xylem flow and shoot-to-root phloem flow (Robert and Friml, 2009). Root-synthesized tZ-type cytokinins move acropetally via the xylem and regulate shoot development and stress response (Ghanem et al., 2011; Ko et al., 2014; Zhang et al., 2014). In contrast, shoot-synthesized iP-type cytokinins move basipetally via the phloem and regulate root development and nodulation (Kudo et al., 2010; Bishopp et al., 2011; Sasaki et al., 2014; Lacombe and Achard, 2016). Therefore, the iP- and tZ-type cytokinins are directionally translocated and transmit different biochemical messages between the underground and aboveground plant organs in response to developmental cues and environmental stimuli (Kiba et al., 2013; Ko and Helariutta, 2017; Osugi et al., 2017).

Phytohormone transporters are involved in long-distance and short-range transport. Over the years, purine permeases (PUPs) and equilibrative nucleoside transporters (ENTs) have been the focus of cytokinin transporter screening (Kang et al., 2017), and Arabidopsis PUP14 and ENT8 as well as rice PUP4 and PUP7 have been identified as transporters involved in cytokinin translocation in planta (Sun et al., 2005; Qi and Xiong, 2013; Zurcher et al., 2016; Xiao et al., 2018). Specifically, AtPUP14 was identified as a cytokinin importer participating in intracellular cytokinin homeostasis (Zurcher et al., 2016). Recently, ATP binding cassette (ABC) transporters were identified to be essential for routing phytohormones, not only within the plant body but also toward the environment (Borghi et al., 2015). AtABCG14 is an essential efflux pump for xylem loading and shootward tZ-type cytokinin translocation (Ko et al., 2014; Zhang et al., 2014; Borghi et al., 2015). The long-distance translocation mediated by AtABCG14 seems to be conserved in planta, as its rice (*Oryza sativa* L.) homolog OsABCG18 controls xylem loading of root-synthesized cytokinins (Zhao et al., 2019). In the shoot, acropetally translocated cytokinins must be unloaded from the xylem and distributed to the target cells via short-range cellular transport. However, the mechanisms underlying the unloading and distribution of the root-synthesized cytokinins remain unknown.

The expression of *AtABCG14* in aerial tissues (Zhang et al., 2014) indicated that this gene may play critical roles in the shoot. Here, using pharmacological methods, grafting, phytohormone profiling, stable isotope tracer, and genetic complementation, we found that Arabidopsis synchronizes the xylem loading of cytokinins in the root and their distribution in the shoot via the phloem by adjusting the tissue-specific expression pattern of *AtABCG14*. Thus, we revealed a mechanism by which AtABCG14 expressed in the shoot participates in the shoot distribution of root-synthesized cytokinins via phloem unloading in Arabidopsis.

## Results

### Expression pattern of *AtABCG14* in aerial tissues

**Figure 1:**
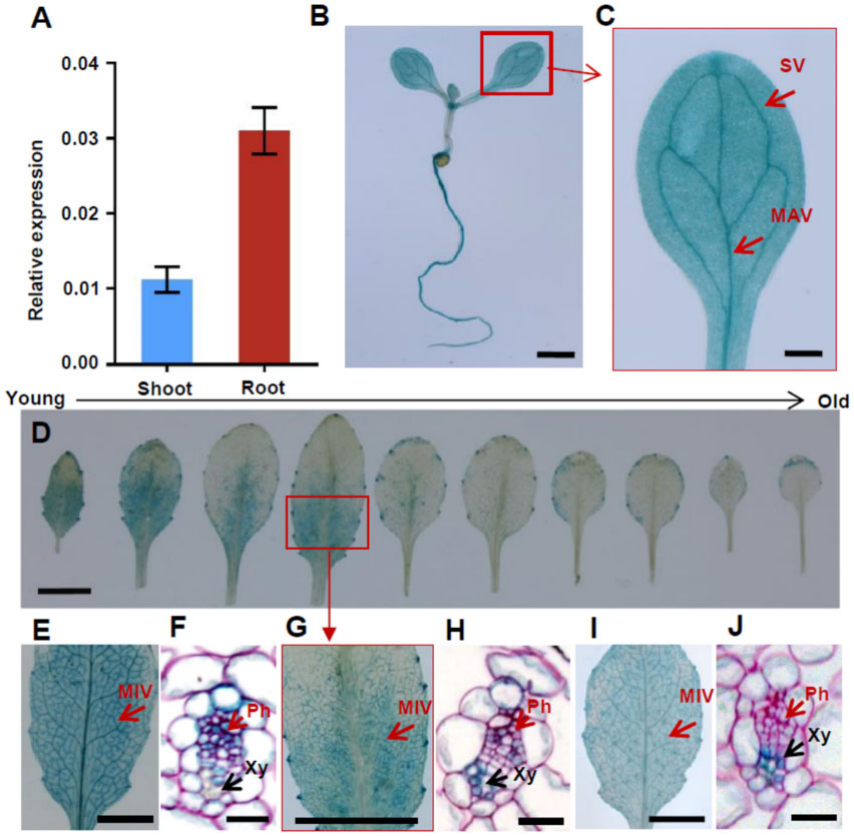
*AtABCG14* expression in aerial tissues. A, *AtABCG14* expression in the shoots and roots of WT seedlings. Data are presented as mean ± standard deviation (sd) (*n* = 4; biological replicates). The transcript level of the target gene was normalized to that of *ACTIN2*. B, GUS staining of transgenic *ABCG14_pro_::GUS* seedlings. Scale bar = 1 mm. C, High-magnification image of single cotyledon in (A). MAV, major vein; SV, secondary vein. Scale bar = 0.2 mm. D, GUS staining of rosette leaves of transgenic *ABCG14_pro_::GUS* plants*.* Younger to older leaves listed from left to right. High-magnification images show that *GUS* is mainly expressed in minor veins of young and growing leaves. E, GUS staining of the seventh rosette leaves in representative transgenic *SUC2_pro_::GUS* plants. F, Ruthenium red-stained cross-sections of the minor veins of transgenic *SUC2_pro_::GUS* plants*.* G, GUS staining of the seventh rosette leaves of transgenic *ABCG14_pro_::GUS* plants. H, Ruthenium red-stained cross-sections of the minor veins of transgenic *ABCG14pro::GUS* plants. I, GUS staining of the seventh rosette leaves in representative transgenic *4CL1_pro_::GUS* plants. J, Ruthenium red-stained cross-sections of the minor veins of transgenic *4CL1_pro_::GUS* plants. MIV, minor veins. Ph, phloem; Xy, xylem. D, E, G, and I, scale bar = 5 mm. F, H and J, scale bar = 30 µm.

We measured *AtABCG14* expression in the shoots and roots of Arabidopsis seedlings. At 10 d after germination (DAG), shoot *AtABCG14* expression was ∼36% of that in the root (Figure 1A). This finding was consistent with β-glucuronidase (GUS) staining and green fluorescence protein (GFP) signals in the shoots of 10-DAG transgenic Arabidopsis seedlings harboring either *ABCG14_pro_::GUS* (Figure 1B) or *ABCG14_pro_::GFP* (Supplemental Figure S1). Cotyledonary GUS staining and GFP signals were detected primarily in the veins (Figure 1C and Supplemental Figure S1). The GFP signal driven by the *ABCG14* promoter overlapped the mCherry signal driven by the phloem companion cell (PCC)-specific *SUCROSE-PROTON SYMPORTER 2* (*SUC2*) promoter (Truernit and Sauer, 1995; Stadler and Sauer, 2019) in dual reporter gene-transgenic Arabidopsis (Supplemental Figure S1). In adult Arabidopsis, *AtABCG14* was expressed mainly in the minor veins of young leaves but only slightly expressed in the margins of old leaves (Figure 1D). A cross-section revealed that *GUS* expression in the minor veins of the young leaves of *ABCG14_pro_::GUS* transgenic plants overlapped with *SUC2_pro_::GUS* expression in the phloem and *4-COUMARATE–COA LIGASE 1* (*4CL1) _pro_::GUS* expression in the xylem (Figure 1, E–J). These results suggest that *AtABCG14* is expressed in the phloem companion and xylem parenchyma cells. In addition, we detected higher tZ-type cytokinin accumulation in the young leaves than in the mature leaves of both wild-type (WT) and *atabcg14* mutant plants. This finding is consistent with the high *AtABCG14* expression in young leaves (Supplemental Figure S2). The unique expression pattern of *AtABCG14* in the aerial tissues indicates that this gene may have a physiological function in the shoot.

### Reciprocal grafting showed that *AtABCG14* plays a critical role in shoot growth

**Figure 2:**
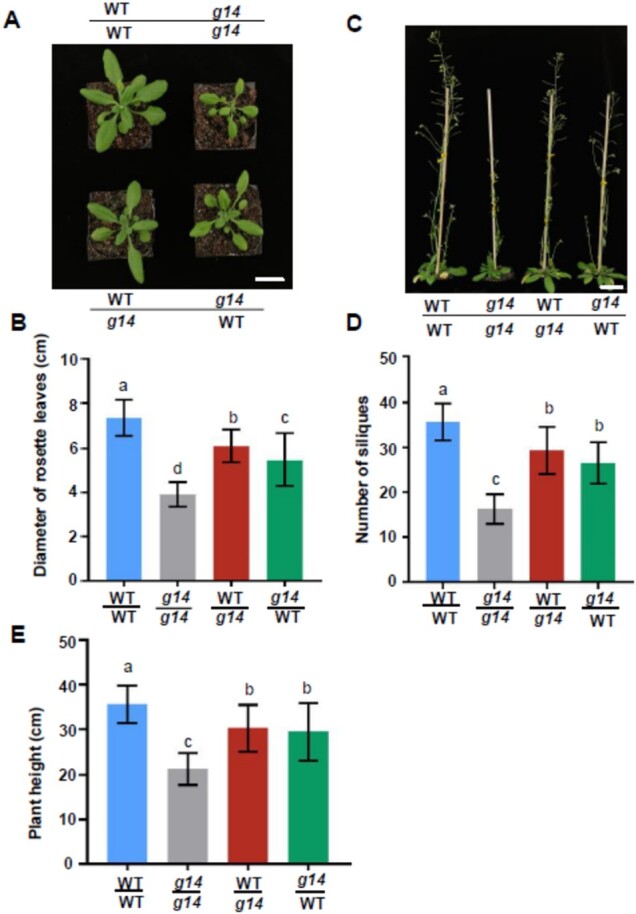
*AtABCG14* disruption in the aerial tissues affects plant growth. A, Phenotypes of rosette leaves of grafted plants at 25 DAG. Scale bar = 1 cm. WT/WT, self-grafted WT; WT/*g14*, WT as scion and *atabcg14* mutant as rootstock; *g14*/*g14*, self-grafted *atabcg14* mutant; *g14*/WT, *atabcg14* mutant as scion and WT as rootstock. B, Diameters of the rosette leaves of grafted plants at 25 DAG. Data are shown as mean ± sd (*n* = 38 independent plants); (C) Phenotypes of entire grafted plants at 50 DAG. Scale bar = 2 cm. D, Number of siliques in grafted plants at 50 DAG. Data are presented as mean ± sd (*n* = 30 independent plants). E, Height of the grafted plants at 50 DAG. Data are presented as means ± sd (*n* = 33 independent plants). Statistical analyses were performed using the one-way analysis of variance (LSD test) with SPSS software (v. 13.0). Different letters above each column indicate significant differences (*P *<* *0.05).

A micro-grafting experiment was performed to assess the specific function of *AtABCG14* in the shoot. We reciprocally grafted WT and *atabcg14* seedlings and monitored shoot growth in the grafted plants (Figure 2). The shoots in the heterografts with WT as the scion and *atabcg14* as the rootstock (WT/*atabcg14*) and in the grafts with *atabcg14* as the scion and WT as the rootstock (*atabcg14*/WT) were smaller than those in the WT/WT homograft (Figure 2, A and B). The diameter of the rosette leaves in the *atabcg14*/*atabcg14* homograft and the *atabcg14*/WT and WT/*atabcg14* heterografts was ∼45%, 25%, and 17% smaller, than that of the WT/WT homograft, respectively (Figure 2, A and B). Moreover, the diameter of the rosette leaves of *atabcg14*/WT was ∼10% smaller than that of WT/*atabcg14* (Figure 2, A and B).

Shoot cytokinins affect seed number in Arabidopsis (Bartrina et al., 2011). The number of siliques in *atabcg14*/*atabcg14*, *atabcg14*/WT, and WT/*atabcg14* was ∼54%, 26%, and 18% lower, than those of WT/WT, respectively. Plants lacking root and/or shoot *AtABCG14* exhibited relatively lower shoot apical meristem (SAM) activity than WT plants (Figure 2, C and D). At maturity, the heterografts *atabcg14*/WT and WT/*atabcg14* were 18% and 15% shorter, than the WT/WT, respectively (Figure 2E). Thus, the WT root can only partially rescue the mutant *atabcg14/*WT phenotype to WT, and *AtABCG14* expression in the aerial tissues is required for shoot growth.

### Cytokinin signaling in the shoot of reciprocally grafted plants between WT and *atabcg14*

**Figure 3:**
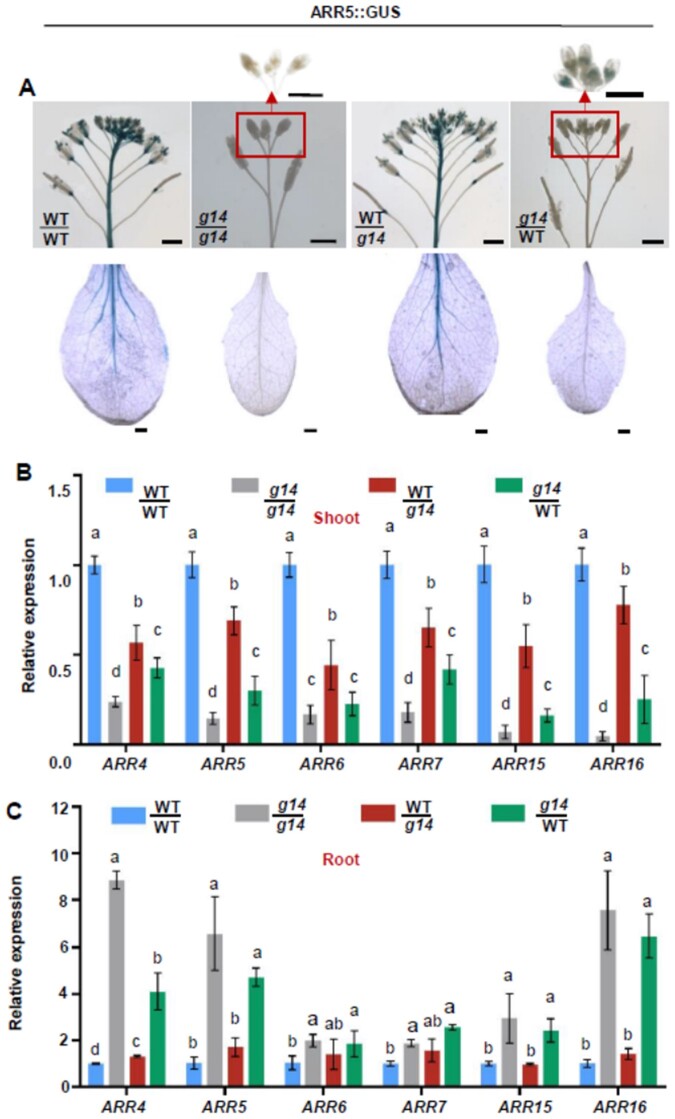
AtABCG14 disruption in the shoot reduces cytokinin signaling. A, GUS staining of flower inflorescence (top) and rosette leaves (bottom) of grafted plants WT/WT, *atabcg14*/*atabcg14*, WT/*atabcg14*, and *atabcg14*/WT at 35 DAG. Scale bar = 1 mm. B and C, Expression levels of type-A cytokinin response genes (*ARR4*, *ARR5*, *ARR6*, *ARR7*, *ARR15*, and *ARR16*) in the shoots (B) and roots (C) of the grafted plants at 25 DAG. The transcript level of the target gene was normalized to that of *ACTIN2*. Data are presented as mean ± sd (*n* = 4 biological replicates; representative data from two independent experiments). Statistical analyses were performed using the one-way analysis of variance (LSD test) with SPSS software (v. 13.0). Different letters above each column indicate significant differences (*P *<* *0.05).

We used GUS staining, GFP fluorescence, and/or reverse transcription quantitative PCR (RT-qPCR) to assay the expression levels of the genes associated with cytokinin signaling in the aerial tissues of the grafted plants (Figure 3). We reciprocally grafted WT and *atabcg14* harboring a cytokinin reporter construct (*ARR5::GUS*) with a *GUS* gene driven by the promoter of *ARABIDOPSIS RESPONSE REGULATOR 5* (*ARR5*) gene. The *atabcg14*/WT graft displayed GUS staining in the flower buds but not the leaves or inflorescences (Figure 3A). In contrast, the same organs exhibited strong GUS staining in the WT/*atabcg14* graft but to a lesser degree than those of the self-grafted WT (Figure 3A). We also grafted plants between WT and *atabcg14* harboring another cytokinin reporter construct (*ARR5::eGFP*) with an *enhanced GFP* (*eGFP*) gene driven by the promoter of *ARR5* gene (Supplemental Figure S3A). GFP fluorescence was intense in the self-grafted WT but absent in the self-grafted *atabcg14*. GFP florescence was substantially reduced in the leaf epidermal cells of the heterograft WT/*atabcg14* and virtually undetectable in those of the heterograft *atabcg14*/WT.

We performed a RT-qPCR assay to quantify the expression of several cytokinin *ARR* genes (D′Agostino et al., 2000) in the grafted inflorescences and rootstocks (Figure 3, B and C). *ARR* expression in the WT/*atabcg14* shoot was partially rescued (range: 28%–73%) but only slightly rescued in the *atabcg14*/WT (range: 6%–24%; Figure 3B). *ARR* expression was considerably higher in the WT/*atabcg14* shoot than in the *atabcg14*/WT shoot (Figure 3B). Contrarily, *ARR* expression was largely rescued in the WT/*atabcg14* root but not in the *atabcg14*/WT root. *ARR* expression was significantly increased (range: 0.9–5.5 folds) in the *atabcg14*/WT root compared with that in the WT/WT root (Figure 3C). Thus, shoot cytokinin signaling was considerably rescued to WT/WT in WT/*atabcg14* but only slightly rescued to WT/WT in *atabcg14/*WT. The considerable reduction in shoot cytokinin signaling in the *atabcg14*/WT indicated that shoot *AtABCG14* expression is a prerequisite for the signaling functions of cytokinins transported from the root to the shoot.

### Long-distance root-synthesized cytokinin transport in plants reciprocally grafted between WT and *atabcg14*

**Figure 4:**
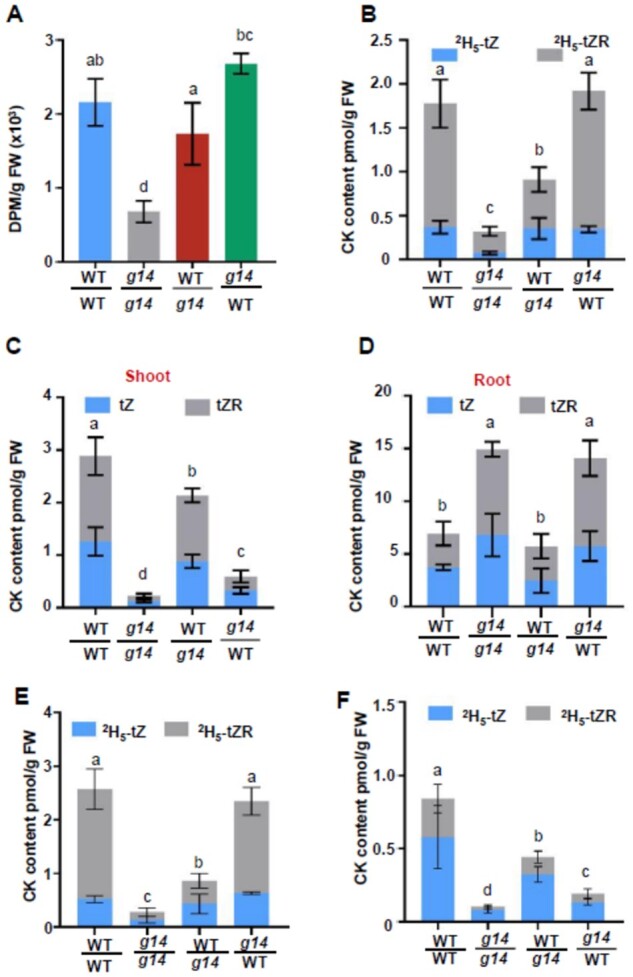
Long-distance translocation of root-synthesized cytokinins in different grafts. A, Radioactivity in the shoots of grafted plants WT/WT, *atabcg14/atabcg14*, WT*/atabcg14*, and *atabcg14/*WT after ^14^C-tZ feeding to the roots for 3 h. B, Quantification of ^2^H_5_-labeled tZ and tZR in the shoots of grafted plants WT/WT, *atabcg14/atabcg14*, WT*/atabcg14*, and *atabcg14/*WT after ^2^H_5_-tZ feeding to the roots for 3 h. C, Endogenous tZ and tZR in the shoots of grafted plants WT/WT, *atabcg14/atabcg14*, WT*/atabcg14*, and *atabcg14/*WT. D, Endogenous tZ and tZR in roots of grafted plants WT/WT, *atabcg14/atabcg14*, WT*/atabcg14*, and *atabcg14/*WT. Data in (A) to (D) are means ± sd (*n* = 3, biological replicates, representative data from two independent experiments). E, Quantification of ^2^H_5_-labeled tZ and tZR in shoots of WT/WT, *atabcg14*/*atabcg14*, WT/*atabcg14*, and *atabcg14*/WT after feeding ^2^H_5_-tZ to roots for 6 h. F, Quantification of ^2^H_5_-labeled tZ and tZR in the shoots of the grafted plants. The roots were fed ^2^H_5_-tZ (5 mM MES; pH 5.7) for 3 h and incubated in ^2^H_5_-tZ-free mock solution for another 3 h. Data are expressed as mean ± sd (*n* = 3 biological replicates). Statistical analyses were performed using the one-way analysis of variance (LSD test) with SPSS software (v. 13.0). Different letters above each column indicate significant differences (*P* < 0.05).

To determine whether root-synthesized cytokinins move to the aerial tissues of various grafts and particularly in *atabcg14*/WT, we fed ^14^C-labeled tZ to the root for 3 h and assayed shoot radioactivity by scintillometry. The shootward ^14^C-labeled tZ transport was suppressed in self-grafted *atabcg14* but rescued in the WT/*atabcg14* and *atabcg14*/WT heterografts compared with that in self-grafted WT (Figure 4A). To determine the extent to which the tZ-type cytokinins delivered to the shoot were intact, we fed the roots of various grafts ^2^H-labeled tZ, which could be converted to tZR (Zhang et al., 2014; Figure 4B). The quantification of ^2^H-labeled tZ and tZR by liquid chromatography-mass spectrometry (LC–MS) revealed that distribution patterns of tZ and tZR were similar to those of ^14^C-labeled tZ (Figure 4B). Root *AtABCG14* expression restored labeled tZ transport in the *atabcg14/*WT grafts. However, the *atabcg14/*WT plants were deficient in cytokinin signaling and their growth was delayed (Figures 2 and 3). Hence, AtABCG14 disruption in the aerial tissues suppressed long-distance root-synthesized cytokinin translocation even after xylem loading was rescued. Moreover, AtABCG14 plays a pivotal role in shoot cytokinin distribution. Labeled tZ and tZR were restored in WT/*atabcg14* with an *AtABCG14-*deficient rootstock (Figure 4, A and B). Thus, an alternative cytokinin xylem-loading pathway independent of AtABCG14 is active in Arabidopsis roots. To quantify endogenous cytokinin accumulation in the grafts, we measured tZ, tZR, IP, IPR, cZ, and cZR in the shoots and roots by LC–MS (Figure 4, C and D; Supplemental Figure S4, A and B). The tZ-type cytokinins were detected mainly in the WT/WT shoots but substantially reduced in *atabcg14*/*atabcg14* (Figure 4C). In addition, the concentration of tZ and tZR was approximately 9 times higher in the WT/*atabcg14* shoots than in the *atabcg14*/*atabcg14* shoots (Figure 4C), and was rescued to 74% of the WT/WT. The concentration of tZ and tZR in the *atabcg14*/WT shoots was increased by approximately 2.5 times compared with that in *atabcg14*/*atabcg14* (Figure 4C). However, these concentrations were still considerably lower than those in WT/WT. The concentration of iP-type cytokinins had increased in *atabcg14*/*atabcg14* and substantially increased in *atabcg14*/WT (Supplemental Figure S4A). Therefore, iP-type cytokinin overaccumulation could compensate for the shortage of tZ-type cytokinins in the shoot. Overall, the cytokinin concentration in the grafted plants was consistent with shoot cytokinin signaling (Figure 3).

Our ^14^C-tZ and ^2^H_5_-tZ assays showed that tZ transport was fully rescued in *atabcg14*/WT and partially rescued in WT/*atabcg14* (Figure 4, A and B). However, the endogenous tZ/tZR was substantially reduced in the *atabcg14*/WT shoots (Figure 4C). To explain the inconsistency, we fed ^2^H_5_-tZ to the roots of various grafts for 3 h, transferred half the plants to mock solution, and incubated them for another 3 h. The cytokinins in the grafted plant shoots were quantified. The labeled tZ-type cytokinin concentrations in the *atabcg14*/WT fed ^2^H_5_-tZ solution were similar to those in WT/WT (Figure 4E). Nevertheless, the labeled tZ-type cytokinin concentrations were considerably lower in *atabcg14*/WT fed mock solution than in WT/WT (Figure 4F), and this is consistent with the endogenous tZ/tZR concentrations (Figure 4C). Therefore, we speculated that the cytokinins in the shoot might be retrograded to the root when AtABCG14 was dysfunctional in the *atabcg14*/WT shoots.

To provide direct evidence of the retrograde transport of root-synthesized tZ-type cytokinins to the roots, we performed a split-root experiment in which the roots of a plant were divided into two groups; one group was immersed in isotope-labeled ^2^H_5_-tZ. The ^2^H_5_-tZ-type cytokinins in the shoot and the other group were quantified and the results showed that ^2^H_5_-tZ/tZR could be transported from the roots to shoots and then retrograded to the roots (Supplemental Figure S5, A–C). This finding directly supports the retrograde transport of root-synthesized tZ-type cytokinins in Arabidopsis.

The root tZ-type cytokinin concentrations in WT/*atabcg14* were similar to those in WT/WT (Figure 4D). This finding was consistent with the results of the isotope tracer experiments (Figure 4, A and B). However, the tZ-type cytokinins hyperaccumulated in the *atabcg14*/WT roots, as in the *atabcg14*/*atabcg14* roots (Figure 4D). The cytokinin concentrations in the grafted plants were consistent with root cytokinin signaling (Figure 3C). We speculated that root-synthesized cytokinin distribution and shootward transport might have been impaired and only a small quantity of tZ-type cytokinin was retained in the *atabcg14*/WT shoot. This indicated that the root-synthesized cytokinins might have been trapped in the *atabcg14*/WT leaf veins and retrograded to the roots.

### Distribution of root-synthesized cytokinins in the shoots of plants reciprocally grafted between WT and *atabcg14*

**Figure 5:**
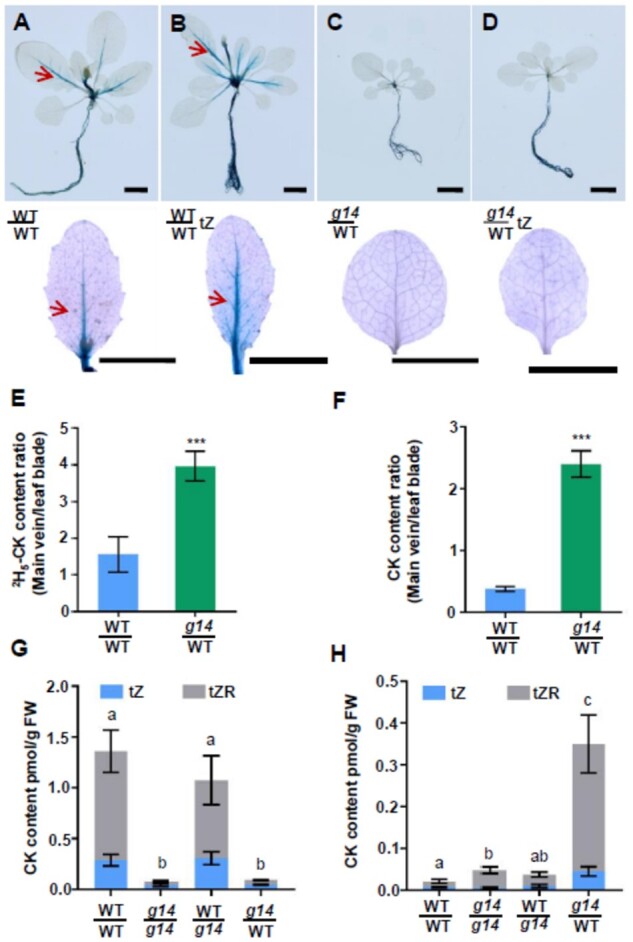
Phloem transport is required to distribute root-synthesized cytokinins in the shoot. A–D, GUS staining of *ARR5::GUS* transgenic grafted plants WT/WT and *atabcg14*/WT (*g14*/WT) after mock and exogenous tZ treatments of the roots. WT/WT and WT/WT (tZ) represent mock-treated (**A**) and tZ-treated (B) self-grafted WT, respectively, for 3 h. *g14*/WT and *g14*/WT (tZ) represent mock-treated (C) and tZ-treated (D) grafted *atabcg14*/WT plants, respectively, for 3 h. Scale bars = 1 cm. E, Ratios of ^2^H_5_-labeled tZ-type cytokinin in the main veins and leaf blades of WT/WT and *atabcg14*/WT. F, Ratios of endogenous tZ-type cytokinins in the major veins and leaf blades of WT/WT and *atabcg14*/WT. Data in (E) and (F) are presented as means ± sd (*n* = 3, biological replicates); ***P <* *0.001 (Student’s *t* test). G, Cytokinin content in the apoplastic extracts of WT/WT, *g14*/*g14*, WT/*g14*, and *g14*/WT grafted plants. H, Cytokinin content in the phloem saps of WT/WT, *g14*/*g14*, WT/*g14*, and *g14*/WT grafted plants. Data in (G) and (H) are presented as mean ± sd (*n* = 3, biological replicates and representative of two independent experiments). Statistical analyses were performed using the one-way analysis of variance (LSD test) with SPSS software (v. 13.0). Different letters above each column indicate significant differences (*P *<* *0.05). The significant differences shown in (G) and (H) refer to the total content of tZ and tZR

To trace root-synthesized cytokinin retention in *atabcg14*/WT leaf veins, we fed exogenous tZ to the roots of various grafts harboring the ARR5::GUS reporter and tracked tZ transport and signaling. Three hours after feeding exogenous tZ to the roots, tZ-type cytokinins were delivered to the shoots (Supplemental Figure S6). *ARR5* expression was measured by GUS staining in the treated plants (Figure 5, A–D). Mock-treated WT/WT showed weak GUS staining near the petioles and midribs (Figure 5A). After root tZ feeding, the GUS signal spread to the laminae (Figure 5B). Hence, cytokinins were successfully delivered to the target cells and induced a signaling cascade there. However, mock-treated (Figure 5C) and tZ-treated *atabcg14*/WT (Figure 5D) failed to induce foliar *GUS* expression. This result and the aforementioned observations (Figure 4, A and B) indicated that root-fed cytokinins could be delivered to the shoot but did not correctly distribute to the target cells or trigger signaling. We repeated the previous experiment on *ARR5::eGFP* transgenic plants to test root-synthesized CK signaling in various live grafts. Consistent with the previous data, we found the GFP signal was nearly rescued in the WT/*abcg14* background but not in *abcg14*/WT (Supplemental Figure S3, A and C). Furthermore, the GFP signals substantially increased in the WT/WT and WT/*abcg14* shoots after the roots were fed tZ (Supplemental Figure S3, A–C). This finding was consistent with the data presented in Figure 5, A–D.

To confirm abnormal root-synthesized cytokinin distribution in *atabcg14*/WT, we subjected WT/WT and *atabcg14*/WT roots with ^2^H_5_-labeled tZ and extracted cytokinins from the midribs and laminae (Figure 5, E and F). The quantification of ^2^H_5_-labeled tZ and endogenous tZ-type cytokinins (Supplemental Table S1) showed that the petiole-midrib:lamina tZ-type cytokinin ratios were considerably higher in *atabcg14*/WT than in WT/WT. These results demonstrated that both the ^2^H_5_-labeled and endogenous tZ-type cytokinins were retained in the midribs and petioles and that their distribution from the veins to the laminae of *atabcg14*/WT was severely impaired.

To identify the recipient (xylem and phloem) cells of root-synthesized cytokinins, we profiled the phytohormones in apoplastic xylem and cell wall extracts (He et al., 2020) and in phloem sap (Tetyuk et al., 2013). The concentration of tZ-type cytokinins was relatively low in the apoplastic extracts of *atabcg14*/*atabcg14* and *atabcg*14/WT. In contrast, the concentration of tZ-type cytokinins was relatively higher in in WT/WT and WT/*atabcg14* apoplastic extracts (Figure 5G). The concentration of tZ-type cytokinins in the phloem sap of *atabcg14*/WT was ≤16.6-fold higher than that in the other three grafts (Figure 5H). For this reason, the tZ-type cytokinins were retained in the leaf vein phloem of *atabcg14*/WT. This finding validates the cytokinin efflux function of *AtABCG14* expressed in PCCs (Figure 1, E–H; Supplemental Figure S1). Abscisic acid (ABA) served as a negative control, and its distribution did not significantly differ among grafts (Supplemental Figure S7). These results collectively showed that *AtABCG14* expression is responsible for the phloem unloading of root-synthesized cytokinins to apoptotic cells.

### Phloem-specific expression of *AtABCG14* rescued the phloem unloading of root-synthesized cytokinins in *atabcg14* shoots

**Figure 6:**
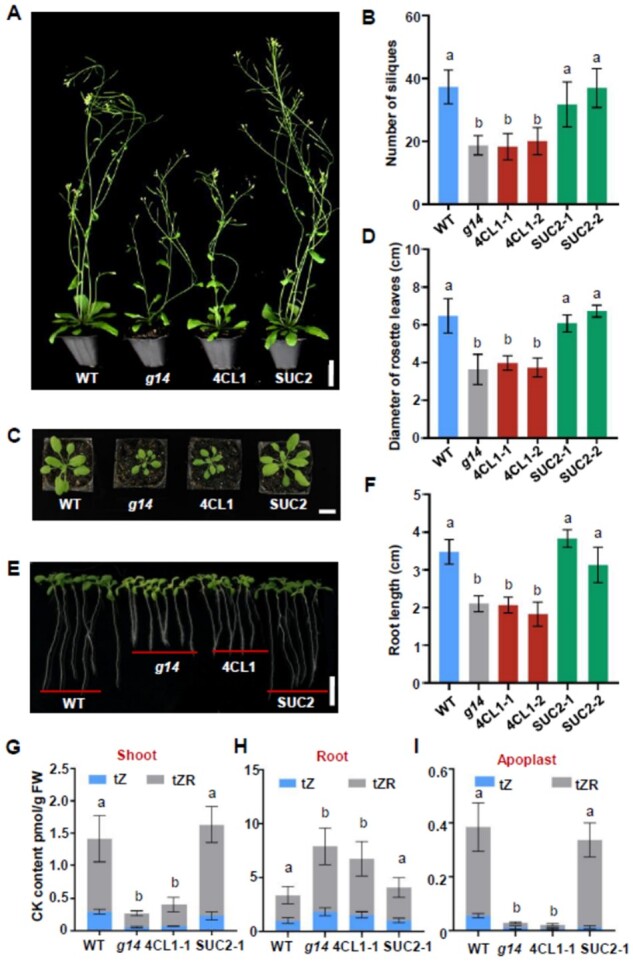
*AtABCG14* expression in the phloem is essential for its function in aerial tissues. A, Phenotypes of 35-DAG transgenic plants of *atabcg14* complemented with *AtABCG14* under xylem-specific promoter 4CL1_pro_ and phloem-specific promoter SUC2_pro_. Scale bar = 3 cm. B, Quantification of silique number of plants in (A). Data are means ± sd (*n* = 9). C, Phenotype of the rosette leaf of *atabcg14* complemented with *AtABCG14* under xylem-specific promoter 4CL1_pro_ and phloem-specific promoter SUC2_pro_. Scale bar = 2 cm. D, Quantification of diameter of rosette leaves in (C). Data are presented as mean ± sd (*n* = 8). E, Root phenotype of 10-DAG seedlings of plants in (A) at T3 generation. Scale bar = 1 cm. F, Quantification of root length of seedlings in (E). Data are presented as mean ± sd (*n* = 20). G–I, Cytokinin content in the shoot (G, *n* = 4), root (H, *n* = 4), and apoplastic extracts (I, *n* = 3) of *atabcg14* complementary lines with *AtABCG14* under xylem-specific promoter 4CL1_pro_ and phloem-specific promoter SUC2_pro_. Data in (G) and (H) are presented as mean ± sd (*n* = 4, biological replicates). Data in (I) are presented as mean ± sd (*n* = 3, biological replicates). *g14* represents *atabcg14*. 4CL1 and SUC2 indicate transgenic plants of *atabcg14* complementary with *AtABCG14* under 4CL1_pro_ and SUC2_pro_ promoters. Statistical analyses were performed using the one-way analysis of variance (LSD test) with SPSS software (v. 13.0). Different letters above each column indicate significant differences (*P *<* *0.05). The significant differences shown in (G) to (I) refer to the total content of tZ and tZR.

*AtABCG14* expression was detected in the phloem and xylem of minor veins (Figure 1, E–J). We sought to determine whether phloem- or xylem-specific *AtABCG14* expression in the aerial tissues is associated with its role in regulating cytokinin distribution. We used the xylem-specific promoter 4CL1_pro_ (Booker et al., 2003) and the PCC-specific promoter SUC2_pro_ (Truernit and Sauer, 1995) to express *AtABCG14* in the *atabcg14* background. The root length, rosette leaf diameter, and silique number of *atabcg14* were substantially different from those of WT (Ko et al., 2014; Zhang et al., 2014). Thus, all three phenotypes were examined to establish the complementary roles of *AtABCG14* driven by various tissue-specific promoters (Figure 6). *AtABCG14* expression under SUC2_pro_ complemented the defective *AtABCG14* phenotypes of *atabcg14*, whereas *AtABCG14* expression driven by 4CL1_pro_ did not (Figure 6, A–F). Cytokinin profiling showed that cytokinin distribution was restored in the transgenic plants wherein *AtABCG14* expression was driven by SUC2_pro_ but not 4CL1_pro_ (Figure 6, G and H; Supplemental Figure S8, A and B). Apoplastic extract assays revealed that *AtABCG14* expression driven by the *SUC2* promoter rescued the apoplastic cytokinins in *atabcg14* leaves (Figure 6I;Supplemental Figure S8C). Grafting experiments between *SUC2_pro_::AtABCG14* transgenic *atabcg14* plants and *atabcg14* mutants showed that *AtABCG14* expression driven by the *SUC2* promoter in the shoot substantially rescued the *atabcg14* growth-retardation phenotype (Supplemental Figure S9). These results showed that *AtABCG14* expression in the PCCs of aerial tissues is essential for the long-distance root-to-shoot transport, distribution, and function of root-synthesized cytokinins in the shoots.

## Discussion

**Figure 7:**
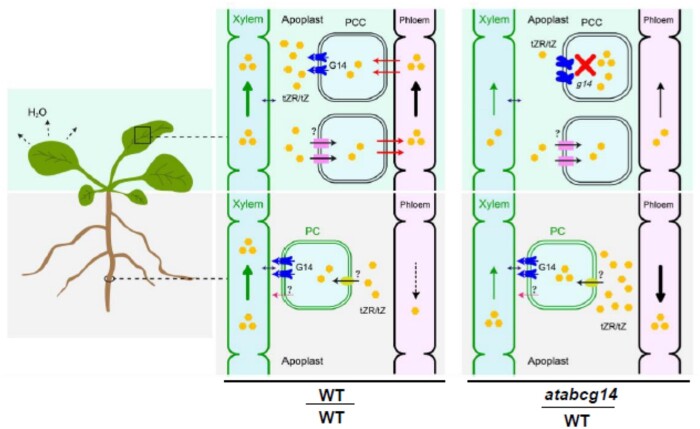
Schematic representation of AtABCG14 in the root loading and shoot distribution of root-synthesized cytokinins in Arabidopsis. tZR/tZ is produced in pericycle cells (PCs) or retrieved from apoplast and loaded into root xylem by G14 (AtABCG14) or via an unknown pathway. Cytokinins are transported via the xylem by the leaf transpiration stream and loaded into the shoot or main vein phloem by an unknown transporter. In self-grafted WT, most cytokinins are transported from the source to the minor veins via the phloem and released into the apoplast by AtABCG14 on PCCs. Small amounts of cytokinins in the phloem may be recycled to the root. In the heterograft *atabcg14*/WT, phloem unloading is suppressed and the majority of root-synthesized cytokinins in the phloem may be retrograded to the roots. tZ, trans-zeatin; tZR, tZ-riboside; G14, AtABCG14; PCC, phloem companion cells; PC, pericycle cells; ?, unknown mechanism.

Various long-distance signaling molecules play essential roles in root-to-shoot communication and coordinate growth and development in response to environmental fluctuations (Ko and Helariutta, 2017). Long-distance translocation events of auxins, ABA, cytokinins, gibberellins, and their precursors have been described (Hartung et al., 2002; Aloni et al., 2005; Regnault et al., 2015, 2016; Tal et al., 2016). Some phytohormone transporters have been characterized but their mechanisms are poorly understood. Root-synthesized tZ-type cytokinins are root-to-shoot signals vital to shoot growth. Here, we elucidated a mechanism by which Arabidopsis uses tissue-specific expression of the transporter AtABCG14 to synchronize xylem loading and phloem unloading of root-synthesized cytokinins (Figure 7).

*AtABCG14* expression in the vascular tissues of shoots and roots enables the synchronization of root loading and shoot distribution of root-synthesized cytokinins. In the root *AtABCG14* expression is high and localized to pericycle, procambial, and phloem cells (Ko et al., 2014; Zhang et al., 2014). *AtABCG14* expression is high in the veins of young leaves but low in those of old leaves (Figure 1D). Leaf vein cross-sections revealed a high expression of *AtABCG14* in the phloem and xylem parenchyma cells (Figure 1, F and G). *AtABCG14* expression driven by *SUC2* rather than the *4CL1* promoter in *atabcg14* shoots (Figure 6; Supplemental Figures S8 and S9) successfully rescued the mutant phenotypes. Hence, *AtABCG14* must be expressed in the phloem for it to perform the physiological function in the shoots. *AtABCG14* localization to the PCCs coincides with the role of this gene in the shoot distribution of root-synthesized cytokinins.

Grafting experiments and phloem sap profiling revealed that AtABCG14 might be responsible for root-synthesized cytokinin unloading from the phloem to the apoplast. The analysis of the leaf apoplastic extracts of reciprocal grafts between *atabcg14* and WT showed that the tZ-type cytokinin concentration in *atabcg14*/*atabcg14* and *atabcg14*/WT was 94% and 93% lower than that in self-grafted WT, respectively (Figure 5G). A lack of shoot *AtABCG14* expression substantially reduced relative shoot *ARR5* and other *ARR* expression (Figure 3, A and B). The phloem sap assay on various grafts showed that the root-synthesized cytokinin concentration was up to 16 times higher in the leaf phloem of *atabcg14*/WT than in the self-grafted WT (Figure 5H). On the contrary, both cytokinin signaling (Figure 3C) and tZ/tZR quantification (Figure 4D) in *atabcg14*/WT root suggested that the over-accumulated root-synthesized cytokinins in the phloem were retrograded to the root (Figure 7). Moreover, the ^2^H_5_-tZ tracer experiment showed that the tZ-type cytokinins can be retrograded from the shoot to the root (Supplemental Figure S5). A previous study showed that AtABCG14 promotes cytokinin efflux from the cytosol to the cell apoplast (Zhang et al., 2014). Our results suggested that AtABCG14-driven cytokinin efflux from the phloem cytosol to the apoplast is required for appropriate root-synthesized cytokinin distribution and shootward translocation in Arabidopsis.

Although AtABCG14 is known to be involved in the xylem loading of root-synthesized cytokinins in the root, evidence from our experiments indicated the presence of an AtABCG14-independent pathway that also plays an essential role in xylem loading in the absence of root-expressed *AtABCG14*. WT/*atabcg14* lacked root AtABCG14, and its shootward transport of labeled and endogenous tZ-type cytokinins was only weakly compromised. Moreover, the latter was sufficient to rescue most of the phenotypes and signaling compared with self-grafted WT (Figures 3 and 4). The cytokinins in the WT/*atabcg14* shoot apoplast and phloem displayed similar patterns to those in WT/WT (Figure 5, G and H). These findings contradict those of a previous study, in which xylem cytokinin loading via AtABCG14 was indispensable because a WT scion grafted onto an *atabcg14* rootstock demonstrated an *atabcg14-*like phenotype while an *atabcg14* scion grafted onto a WT rootstock demonstrated a WT-like phenotype (Ko et al., 2014). In contrast, our findings are consistent with those of other studies wherein grafts with a WT scion and a rootstock of *ARABIDOPSIS ISOPENTENYLTRANSFERASE 1; 3; 5; 7* quadruple mutant *atipt1;3;5;7* or *cyp735a1* and *cyp735a2* double mutant *cypDM*, respectively, showed WT-like phenotypes (Matsumoto-Kitano et al., 2008; Kiba et al., 2013).

The root-synthesized cytokinin levels in the shoot and leaf apoplasts of the *atabcg14* mutant were 92% and 94% lower than those in the WT, respectively (Figures 4, C and 5, G). Despite its stunted growth, the *atabcg14* mutant had a normal life cycle. Cytokinin signaling in the floral meristem was partially rescued in *atabcg14*/WT (Figure 3A). Thus, a limited amount of root-synthesized cytokinins can be delivered to the SAM in the absence of AtABCG14. AtABCG14-independent phloem unloading might occur via the symplastic transport pathway used to unload sugar and nutrients (Schmalstig and Geiger, 1985; Wang et al., 2012). Considering the above results, we speculate that apoplastic transport is the primary pathway for unloading, whereas symplastic transport likely serves as a secondary pathway maintaining the normal growth of plants.

Nevertheless, it remains unclear how and where root-synthesized cytokinins transmitted in the xylem are exchanged into the phloem. As the xylem is a part of the apoplast and does not require unloading transporters (Kang et al., 2017), putative cytokinin importers on the plasma membranes of phloem companion or xylem parenchyma cells may be required for the xylem-to-phloem transfer. Arabidopsis PUP14 is characterized as a cytokinin importer (Zurcher et al., 2016), and OsENT2 reportedly exhibits iPR transport activity (Hirose et al., 2005). Hence, certain members of the PUP or ENT family might participate in xylem-to-phloem translocation. In summary, we found that phloem unloading is required for the shoot distribution of the root-synthesized cytokinins in Arabidopsis. Moreover, AtABCG14 serves as an exporter in phloem unloading and synchronizes root loading and shoot distribution of root-synthesized cytokinins (Figure 7). This model may represent a generic mechanism for the distribution of other shootward plant growth regulators or nutrients, and this should be explored in the future.

## Materials and methods

### Plant materials and growth conditions

Arabidopsis ecotype Columbia (Col)-4 was used in this study. *atabcg14*, *atabcg14*/*ARR5::GUS*, *atabcg14/ARR5::EGFP, ABCG14_pro_::GUS*, *ARR5::GUS, and ARR5::eGFP* transgenic plants or mutants were described previously (Zhang et al., 2014, 2020). Seeds were sown on Petri dishes containing half-strength Murashige and Skoog (1/2 MS) medium (M519; PhytoTechnology Laboratories, Shawnee Mission, KS, USA), 0.5% (w/v) gellan gum (G434; PhytoTechnology Laboratories, Shawnee Mission, KS, USA), and appropriate antibiotics. They were incubated at 4°C for 3 d before being transferred to a growth chamber. Seedlings with two true leaves were transplanted to soil (Professional growing mix; Sun Gro Horticulture Canada Ltd., Seba Beach, AB T0E 2B0, Canada) under a 16-h light/8-h dark light cycle and ∼60% relative humidity unless otherwise indicated. The light intensity was ∼110 μmol m^−2^ s^−1^. The WT, mutant, and/or transgenic plants were grown simultaneously in the same trays to minimize variations in the growth conditions.

### Gene expression analysis

The total RNA was extracted using the TaKaRa Minibest Universal RNA Extraction Kit (No. 9767; TaKaRa Bio Inc., Kusatsu, Shiga, Japan). The cDNAs were synthesized with HiScript qRT Supermix for qPCR (+gDNA wiper; R123-01; Vazyme Biotech, Nanjing, China). The RT-qPCR was performed using SYBR Green (TaKaRa Bio Inc., Kusatsu, Shiga, Japan) on the ABI PRISM 7700 System (Applied Biosystems, Foster City, CA, USA). *ACTIN2* was used as the internal control for RT-qPCR data analysis. The RT-qPCR primers are listed in Supplemental Table S2. The transcript level of the target genes was normalized to that of *ACTIN2*.

### Plasmid construction

The *mCherry* fragment was amplified using the mCherry-F and mCherry-R primers and then ligated to pMDC163 and digested with XbaI and SacI. Therefore, *GUS* was replaced with *mCherry*, and pMDC163-mCherry was generated. SUC2_pro_-pDONR207 was ligated to pMDC163-mCherry using the LR reaction (11791-020; Invitrogen, Carlsbad, CA, USA) to obtain pMDC163-SUC2_pro_-mCherry. AtABCG14_pro_-pDONR207 was ligated to pMDC107 using the LR reaction (Invitrogen, Carlsbad, CA, USA) to generate pMDC107-ABCG14_pro_-GFP. All primers are listed in Supplemental Table S2.

*AtABCG14* was amplified using primers AtABCG14-P1 and AtABCG14-P2 using the cDNA of WT as the template. After digestion with XbaI and SacI, the fragment was ligated to pMDC163, digested again with XbaI and SacI, and the *GUS* gene was replaced with *AtABCG14* to obtain the pMDC163-ABCG14 plasmid. 4CL_pro_-pDONR207 and SUC2_pro_-pDONR207 were ligated to pMDC163-ABCG14 using the LR reaction (Invitrogen, Carlsbad, CA, USA) to form the pMDC163-4CL_pro_-ABCG14 and pMDC163-SUC2_pro_-ABCG14 plasmids. The plasmids were transformed in *Agrobacterium tumefaciens* strain GV3101 and infiltrated into the *atabcg14* mutant using the floral dip method (Zhang et al., 2006). All primers used are listed in Supplemental Table S2. The promoter regions of *AtABCG14*, *SUC2*, and *4CL1* were 1,295, 2,129, and 1,001 bp in length, respectively.

### Grafting

The micro-graft method used here was modified from a previous protocol (Yin et al., 2012). Briefly, the seeds were grown on slope (slant) medium (1/2 MS, 1.5% (w/v) agar, 3% (w/v) sucrose, 15° angle) for 5 d. Under a dissecting microscope (NSZ-606, Xiamen Phio Scientific Instruments Co., Ltd., Xiamen, China), the seedling hypocotyls were cut vertically in the middle with a sharp razor blade. The scion was quickly moved to the target rootstock, and both scion and rootstock were firmly squeezed together. The Petri dishes were vertically aligned in a growth chamber. One day after surgery, the scion and rootstock were vertically adjusted and matched again. Two days after surgery, the roots and shoots were joined. Samples without an appropriate connection were discarded. Adventitious roots on the scions were removed with a pair of sharp tweezers. Well-grafted seedlings with two true leaves were transplanted to the soil. The cut edges were left above the soil surface and checked daily. Any new adventitious roots were immediately removed once they had formed.

### Cytokinin quantification

Cytokinins were extracted using a previously described method (Zhao et al., 2019). One hundred milligrams of ground tissue was added to each 2-mL plastic microtube (Eppendorf AG, Hamburg, Germany), frozen in liquid nitrogen, and homogenized in a Tissuelyser-48 (Shanghai Jingxin Experimental Technology, Shanghai, China). The ground tissues were mixed with 1 mL of 80% (v/v) methanol with 45 pg of internal standards (i.e. [^2^H_5_]tZ and [^2^H_5_]tZR; OlChemIm s.r.o., Olomouc, Czech Republic) and extracted twice using a laboratory rotator for 2 h at 4°C. After centrifugation (10 min, 15,000*g*, 4°C), the supernatant was collected, transferred to a clean plastic microtube, and dried with nitrogen gas flow. The pellet was dissolved in 300 μL of 30% (v/v) methanol. After centrifugation (10 min, 15,000*g*, 4°C), the supernatant was passed through a 0.22-μm membrane filter and used in cytokinin quantification according to a previously described method (Zhao et al., 2019).

### Tracer experiments

Isotope (^14^C- or ^2^H_5_-tZ) tracer experiments were performed as previously described (Zhang et al., 2014). For the ^14^C-tZ isotope uptake assay, different 12-DAG grafted seedlings were used. The seedling roots were immersed in 5 mM MES-KOH (pH 5.6) buffer containing ^14^C-tZ (3,700 Bq mL^−1^; final concentration 5 nM; American Radiolabeled Chemicals, St Louis, MO, USA) under normal growth conditions. After 3 h of incubation, the roots and shoots were carefully separated with a razor blade at their junction. The shoots from three plants per graft type were separately collected as replicates. The samples were mixed with 1.5 mL of bleach (8.25% (v/v) sodium hypochlorite; Clorox, Oakland, CA, USA) and incubated at 70°C for 3 h to solubilize the tissues. After centrifugation (10 min, 12,000*g*, 4°C), the radioactivity of the supernatant was quantified under a scintillometer (LS 6000; Beckman Coulter, Pasadena, CA, USA). For the ^2^H_5_-tZ isotope uptake assay, the 25-DAG grafted plants were used. The roots were immersed in 5 mM MES-KOH (pH 5.6) buffer containing ^2^H_5_-tZ (final concentration 50 nM; OlChemIm s.r.o., Olomouc, Czech Republic) under normal growth conditions for 3 h. The shoot cytokinins were extracted and quantified according to a previously described method, in which [^2^H_6_]iP (OlChemIm s.r.o., Olomouc, Czech Republic) was used as the internal standard (Zhao et al., 2019).

### GUS staining

Histochemical GUS staining was performed using a previously described method (Jefferson et al., 1987). The samples were infiltrated with 90% (v/v) acetone for 30 min on ice and washed thrice with ultrapure water. Samples were infiltrated under vacuum in GUS staining buffer (0.5 mg mL^−1^ 5-bromo-4-chloro-3-indolyl glucuronide in 0.1 M Na_2_HPO4, pH 7.0, 10 mM Na_2_EDTA, 0.5 mM potassium ferricyanide/ferrocyanide, and 0.1% (v/v) Triton X-100) for 10 min, and then incubated at 37°C for 3 h (Figure 1, B and C) or 12 h (Figures 1, D–G, 3A, and 4, A–D; Supplemental Figure S4). After removal of the staining buffer, the samples were cleared with 70% (v/v) ethanol. Observations under the light stereomicroscope (Discovery V12; Carl Zeiss AG, Oberkochen, Germany) were recorded using a CANON EOS60D camera (Canon Inc., Tokyo, Japan).

For sectioning, the plant tissues were fixed in formalin-acetic acid alcohol (1.8% (v/v) formalin, 5% (v/v) acetic acid, and 90% (v/v) methanol) and dehydrated with an ethanol concentration-gradient series. The liquid was then replaced with pre-infiltration solution (100% ethanol:Technovit 7100 resin base liquid [Kulzer GmbH, Hanau, Germany]; 1:1) and left to stand for 2 h. The samples were transferred to the infiltration solution (1 g hardener I (Kulzer GmbH, Hanau, Germany) in 100 mL of Technovit 7100 base liquid) and incubated for 4 h. The samples were then embedded in infiltration solution by adding 1/15 volume hardener II (Kulzer GmbH, Hanau, Germany) for 4 h at 65°C. The samples were then cut into 4-µm-thick sections using a Leica EM UC7 rotary microtome (Leica Microsystems, Wetzlar, Germany) to observe the leaf minor veins. Images were taken under a BX53 microscope (Olympus, Tokyo, Japan) with a DFC7000 T camera (Leica Camera AG, Wetzlar, Germany).

### SUC2_pro_-mCherry and ABCG14_pro_-GFP co-localization

The pMDC163-SUC2_pro_-mCherry and pMDC107-ABCG14_pro_-GFP were individually transformed in WT using the flower dipping method (Clough and Bent, 1998). Two types of transgenic plants with GFP and mCherry florescence were crossed to generate double-transgenic plants containing pMDC163-SUC2_pro_-mCherry and pMDC107-ABCG14_pro_-GFP. Ten-day-old seedlings harboring pMDC163-SUC2_pro_-mCherry and pMDC107-ABCG14_pro_-GFP were observed under the BX53 fluorescence microscope (Olympus, Tokyo, Japan) fitted with a U-FYFP filter (exciter, 490–500 nm; barrier, 515–560 nm) and a U-FRFP filter (exciter, 535–555 nm; barrier, 570–625 nm) and recorded using DFC7000 T (Leica Camera AG, Wetzlar, Germany).

### Apoplast and phloem sap extraction

Apoplasts were extracted according to a previously described method (He et al., 2020) using the fifth to eighth rosette leaves of 30-DAG grafts. The rosette leaves from two (WT/WT and WT/*atabcg14*) or three (For *atabcg14*/*atabcg14* and *atabcg14*/WT) plants cut at the bases of the laminae, near the petioles, were collected as a sample. The detached leaves were immersed in syringes containing 30 mL of water and the plungers were pulled and pushed repeatedly to facilitate the penetration of water into the apoplasts. After saturation, the leaves were taken out, wrapped carefully with parafilm, and fixed with a 1-mL pipette tip, which was then fastened to a second parafilm strip, suspended above the bottom of a 15-mL centrifuge tube, and centrifuged at 2,500*g* for 10 min at 4°C. The solution was then collected for phytohormone determination according to a previously described method (Zhao et al., 2019).

Phloem sap was extracted according to a previously described method (Tetyuk et al., 2013) using the fifth to eighth leaves from the 30-DAG grafts. The leaves from two (WT/WT and WT/*atabcg14*) or three (*atabcg14*/*atabcg14* and *atabcg14*/WT) plants were cut at the bases of the petioles and near the centers of the rosettes and pooled as one sample. The ends of the petioles were immediately placed in 9-cm Petri dishes containing 20 mM K_2_-EDTA (pH 7.0) and incubated in the dark. The tips of the petiole were completely submerged in the solution. After 1 h, the leaves were gently removed from the Petri dishes, and the tips of the petioles were rinsed thoroughly rinsed with distilled water and immediately placed in water in 1.5-mL plastic tubes in the dark. After 5 h, the exudates were collected for phytohormone detection according to a previously described method (Zhao et al., 2019).

### Statistical analyses

The two-tailed Student’s *t* test (Figure 4, E and F; Supplemental Figure S3) and one-way ANOVA （analysis of variance） were used to analyze differences between plants and/or treatments . In the figures, the different letters above each column indicate significant differences (*P *<* *0.05) according to the LSD （least significant difference） test.

### Accession numbers

Sequence data of most genes assessed in this study can be found in the *Arabidopsis* Genome Initiative database under the following accession numbers: *AtABCG14* (AT1G31770), *AtSUC2* (AT1G22710), *ARR5* (AT3G48100), *ARR4* (AT1G10470), *ARR6* (AT5G62920), *ARR7* (AT1G19050), *ARR15* (AT1G74890), *ARR16* (AT2G40670), and *ACTIN2* (AT3G18780).

## Supplemental data

The following materials are available in the online version of this article.

**Supplemental Figure S1**. *AtABCG14* and *SUC2* co-expression in the veins of Arabidopsis seedlings.

**Supplemental Figure S2**. Quantification of tZ and tZR in the mature and young rosette leaves of WT and *atabcg14* mutant.

**Supplemental Figure S3**. GFP fluorescence of *ARR5::eGFP* transgenic plant in the grafts between WT and *atabcg14* after root tZ treatment.

**Supplemental Figure S4**. Quantification of endogenous cZ, cZR, iP, and iPR in the shoots, roots, phloem saps, and apoplasts of the grafted plants.

**Supplemental Figure S5**. Retrograde transport of tZ-type cytokinins from the shoot to the root.

**Supplemental Figure S6**. Shoot cytokinin concentration in the grafted plants treated with exogenous tZ in roots.

**Supplemental Figure S7**. Abscisic acid concentration in phloem saps and apoplastic extracts of grafted plants.

**Supplemental Figure S8**. Quantification of endogenous cZ, cZR, iP, and iPR in the shoots, roots, and apoplast of *atabcg14* complementary lines with *AtABCG14* under xylem-specific promoter 4CL1_pro_ and phloem-specific promoter SUC2_pro_.

**Supplemental Figure S9**. *AtABCG14* expression driven by SUC2_pro_ in the shoot largely rescued growth-retarded phenotypes of *atabcg14*.

**Supplemental Table S1**. Quantification of cytokinins in leaf midrib and blade of WT/WT and *atabcg14/*WT fed ^2^H_5_-tZ-type cytokinin in roots.

**Supplemental Table S2**. Primers used in this study.

## Supplementary Material

kiab188_Supplementary_Data

## Notes

K.Z., J.Z., C.Z., and C.L. conceived and designed the experiments; K.Z. supervised the study; J.Z., B.D., X.D., and E.Z. conducted the gene expression analysis; J.Z., B.D., X.D., M.Z., P.Z., and L.W. conducted the grafting experiment; E.Z., X.D., B.D., and J.Z. conducted GUS staining and histological sectioning; B.D. conducted the plasmid construction and generated the transgenic plants; J.Z. conducted tracer experiments and the apoplast and phloem sap assays; J.Z., Y.D., and S.X. performed cytokinin profiling; K.Z., J.Z., C.Z., and C.L. analyzed the data and wrote the paper. All authors discussed the results and collectively edited the manuscript.

The author responsible for distribution of materials integral to the findings presented in this article in accordance with the policy described in the Instructions for Authors (https://academic.oup.com/plphys/pages/general-instructions) is: Kewei Zhang (kwzhang@zjnu.edu.cn).

## References

[kiab188-B1] Aloni R , Langhans M, Aloni E, Dreieicher E, Ullrich CI (2005) Root-synthesized cytokinin in Arabidopsis is distributed in the shoot by the transpiration stream. J Exp Bot 56**:** 1535–1544 15824073 10.1093/jxb/eri148

[kiab188-B2] Bartrina I , Otto E, Strnad M, Werner T, Schmulling T (2011) Cytokinin regulates the activity of reproductive meristems, flower organ size, ovule formation, and thus seed yield in Arabidopsis thaliana. Plant Cell 23**:** 69–80 21224426 10.1105/tpc.110.079079 PMC3051259

[kiab188-B3] Bishopp A , Lehesranta S, Vaten A, Help H, El-Showk S, Scheres B, Helariutta K, Mahonen AP, Sakakibara H, Helariutta Y (2011) Phloem-transported cytokinin regulates polar auxin transport and maintains vascular pattern in the root meristem. Curr Biol 21**:** 927–932 21620705 10.1016/j.cub.2011.04.049

[kiab188-B4] Booker J , Chatfield S, Leyser O (2003) Auxin acts in xylem-associated or medullary cells to mediate apical dominance. Plant Cell 15**:** 495–507 12566587 10.1105/tpc.007542 PMC141216

[kiab188-B5] Borghi L , Kang J, Ko D, Lee Y, Martinoia E (2015) The role of ABCG-type ABC transporters in phytohormone transport. Biochem Soc Trans 43**:** 924–930 26517905 10.1042/BST20150106 PMC4613532

[kiab188-B6] Clough SJ , Bent AF (1998) Floral dip: a simplified method for Agrobacterium-mediated transformation of Arabidopsis thaliana. Plant J 16**:** 735–743 10069079 10.1046/j.1365-313x.1998.00343.x

[kiab188-B7] D'Agostino IB , Deruere J, Kieber JJ (2000) Characterization of the response of the Arabidopsis response regulator gene family to cytokinin. Plant Physiol 124**:** 1706–1717 11115887 10.1104/pp.124.4.1706 PMC59868

[kiab188-B8] De Rybel B , Adibi M, Breda AS, Wendrich JR, Smit ME, Novak O, Yamaguchi N, Yoshida S, Van Isterdael G, Palovaara J, et al (2014) Integration of growth and patterning during vascular tissue formation in Arabidopsis. Science 345**:** 1255215 25104393 10.1126/science.1255215

[kiab188-B9] Ghanem ME , Albacete A, Smigocki AC, Frebort I, Pospisilova H, Martinez-Andujar C, Acosta M, Sanchez-Bravo J, Lutts S, Dodd IC, et al (2011) Root-synthesized cytokinins improve shoot growth and fruit yield in salinized tomato (Solanum lycopersicum L.) plants. J Exp Bot 62**:** 125–140 20959628 10.1093/jxb/erq266 PMC2993914

[kiab188-B10] Hartung W , Sauter A, Hose E (2002) Abscisic acid in the xylem: where does it come from, where does it go to? J Exp Bot 53**:** 27–32 11741037

[kiab188-B11] He J , Liu Y, Yuan D, Duan M, Liu Y, Shen Z, Yang C, Qiu Z, Liu D, Wen P, et al (2020) An R2R3 MYB transcription factor confers brown planthopper resistance by regulating the phenylalanine ammonia-lyase pathway in rice. Proc Natl Acad Sci U S A 117**:** 271–277 31848246 10.1073/pnas.1902771116 PMC6955232

[kiab188-B12] Hirose N , Makita N, Yamaya T, Sakakibara H (2005) Functional characterization and expression analysis of a gene, OsENT2, encoding an equilibrative nucleoside transporter in rice suggest a function in cytokinin transport. Plant Physiol 138**:** 196–206 15849298 10.1104/pp.105.060137 PMC1104175

[kiab188-B13] Hirose N , Takei K, Kuroha T, Kamada-Nobusada T, Hayashi H, Sakakibara H (2008) Regulation of cytokinin biosynthesis, compartmentalization and translocation. J Exp Bot 59**:** 75–83 17872922 10.1093/jxb/erm157

[kiab188-B14] Hwang I , Sheen J, Muller B (2012) Cytokinin signaling networks. Annu Rev Plant Biol 63**:** 353–380 22554243 10.1146/annurev-arplant-042811-105503

[kiab188-B15] Jefferson RA , Kavanagh TA, Bevan MW (1987) GUS fusions: beta-glucuronidase as a sensitive and versatile gene fusion marker in higher plants. EMBO J 6**:** 3901–3907 3327686 10.1002/j.1460-2075.1987.tb02730.x PMC553867

[kiab188-B16] Kang J , Lee Y, Sakakibara H, Martinoia E (2017) Cytokinin transporters: GO and STOP in signaling. Trends Plant Sci 22**:** 455–461 28372884 10.1016/j.tplants.2017.03.003

[kiab188-B17] Kiba T , Takei K, Kojima M, Sakakibara H (2013) Side-chain modification of cytokinins controls shoot growth in Arabidopsis. Dev Cell 27**:** 452–461 24286826 10.1016/j.devcel.2013.10.004

[kiab188-B18] Ko D , Helariutta Y (2017) Shoot-root communication in flowering plants. Curr Biol 27**:** R973–R978 28898670 10.1016/j.cub.2017.06.054

[kiab188-B19] Ko D , Kang J, Kiba T, Park J, Kojima M, Do J, Kim KY, Kwon M, Endler A, Song WY, et al (2014) Arabidopsis ABCG14 is essential for the root-to-shoot translocation of cytokinin. Proc Natl Acad Sci U S A 111**:** 7150–7155 24778257 10.1073/pnas.1321519111 PMC4024864

[kiab188-B20] Kudo T , Kiba T, Sakakibara H (2010) Metabolism and long-distance translocation of cytokinins. J Integr Plant Biol 52**:** 53–60 20074140 10.1111/j.1744-7909.2010.00898.x

[kiab188-B21] Lacombe B , Achard P (2016) Long-distance transport of phytohormones through the plant vascular system. Curr Opin Plant Biol 34**:** 1–8 27340874 10.1016/j.pbi.2016.06.007

[kiab188-B22] Liu CJ , Zhao Y, Zhang K (2019) Cytokinin transporters: Multisite players in cytokinin homeostasis and signal distribution. Front Plant Sci 10**:** 693 31214217 10.3389/fpls.2019.00693 PMC6555093

[kiab188-B23] Matsumoto-Kitano M , Kusumoto T, Tarkowski P, Kinoshita-Tsujimura K, Vaclavikova K, Miyawaki K, Kakimoto T (2008) Cytokinins are central regulators of cambial activity. Proc Natl Acad Sci U S A 105**:** 20027–20031 19074290 10.1073/pnas.0805619105 PMC2605004

[kiab188-B24] Osugi A , Kojima M, Takebayashi Y, Ueda N, Kiba T, Sakakibara H (2017) Systemic transport of trans-zeatin and its precursor have differing roles in Arabidopsis shoots. Nat Plants 3**:** 17112 28737742 10.1038/nplants.2017.112

[kiab188-B25] Qi Z , Xiong L (2013) Characterization of a purine permease family gene OsPUP7 involved in growth and development control in rice. J Integr Plant Biol 55**:** 1119–1135 24034337 10.1111/jipb.12101

[kiab188-B26] Regnault T , Daviere JM, Achard P (2016) Long-distance transport of endogenous gibberellins in Arabidopsis. Plant Signal Behav 11**:** e1110661 26515330 10.1080/15592324.2015.1110661 PMC4871640

[kiab188-B27] Regnault T , Daviere JM, Wild M, Sakvarelidze-Achard L, Heintz D, Carrera Bergua E, Lopez Diaz I, Gong F, Hedden P, Achard P (2015) The gibberellin precursor GA12 acts as a long-distance growth signal in Arabidopsis. Nat Plants 1**:** 15073 27250008 10.1038/nplants.2015.73

[kiab188-B28] Robert HS , Friml J (2009) Auxin and other signals on the move in plants. Nat Chem Biol 5**:** 325–332 19377459 10.1038/nchembio.170

[kiab188-B29] Sakakibara H (2006) Cytokinins: activity, biosynthesis, and translocation. Annu Rev Plant Biol 57**:** 431–449 16669769 10.1146/annurev.arplant.57.032905.105231

[kiab188-B30] Santner A , Calderon-Villalobos LI, Estelle M (2009) Plant hormones are versatile chemical regulators of plant growth. Nat Chem Biol 5**:** 301–307 19377456 10.1038/nchembio.165

[kiab188-B31] Sasaki T , Suzaki T, Soyano T, Kojima M, Sakakibara H, Kawaguchi M (2014) Shoot-derived cytokinins systemically regulate root nodulation. Nat Commun 5**:** 4983 25236855 10.1038/ncomms5983

[kiab188-B32] Schmalstig JG , Geiger DR (1985) Phloem unloading in developing leaves of sugar beet: I. evidence for pathway through the symplast. Plant Physiol 79**:** 237–241 16664377 10.1104/pp.79.1.237 PMC1074858

[kiab188-B33] Stadler R , Sauer N (2019) The AtSUC2 promoter: a powerful tool to study phloem physiology and development. Methods Mol Biol 2014**:** 267–287 31197803 10.1007/978-1-4939-9562-2_22

[kiab188-B34] Sun J , Hirose N, Wang X, Wen P, Xue L, Sakakibara H, Zuo J (2005) Arabidopsis SOI33/AtENT8 gene encodes a putative equilibrative nucleoside transporter that is involved in cytokinin transport in planta. J Integr Plant Biol 47**:** 588–603

[kiab188-B35] Tal I , Zhang Y, Jorgensen ME, Pisanty O, Barbosa IC, Zourelidou M, Regnault T, Crocoll C, Olsen CE, Weinstain R, et al (2016) The Arabidopsis NPF3 protein is a GA transporter. Nat Commun 7**:** 11486 27139299 10.1038/ncomms11486 PMC4857387

[kiab188-B36] Tetyuk O , Benning UF, Hoffmann-Benning S (2013) Collection and analysis of Arabidopsis phloem exudates using the EDTA-facilitated Method. J Vis Exp 23: e51111 10.3791/51111 PMC3960974 24192764

[kiab188-B37] Truernit E , Sauer N (1995) The promoter of the Arabidopsis thaliana SUC2 sucrose-H+ symporter gene directs expression of beta-glucuronidase to the phloem: evidence for phloem loading and unloading by SUC2. Planta 196**:** 564–570 7647685 10.1007/BF00203657

[kiab188-B38] Wang YY , Hsu PK, Tsay YF (2012) Uptake, allocation and signaling of nitrate. Trends Plant Sci 17**:** 458–467 22658680 10.1016/j.tplants.2012.04.006

[kiab188-B39] Xiao Y , Liu D, Zhang G, Gao S, Liu L, Xu F, Che R, Wang Y, Tong H, Chu C (2018) Big Grain3, encoding a purine permease, regulates grain size via modulating cytokinin transport in rice. J Integr Plant Biol 61: 581–597 30267474 10.1111/jipb.12727

[kiab188-B40] Yin H , Yan B, Sun J, Jia P, Zhang Z, Yan X, Chai J, Ren Z, Zheng G, Liu H (2012) Graft-union development: a delicate process that involves cell-cell communication between scion and stock for local auxin accumulation. J Exp Bot 63**:** 4219–4232 22511803 10.1093/jxb/ers109 PMC3398452

[kiab188-B41] Zhang K , Novak O, Wei Z, Gou M, Zhang X, Yu Y, Yang H, Cai Y, Strnad M, Liu CJ (2014) Arabidopsis ABCG14 protein controls the acropetal translocation of root-synthesized cytokinins. Nat Commun 5**:** 3274 24513716 10.1038/ncomms4274

[kiab188-B42] Zhang M , Ding B, Zhao J, Zhang P, Li Y, Yang G, Zhang K (2020) A fluorescence-based high-throughput screening method for cytokinin translocation mutants. Plant Methods 16**:** 134 33042209 10.1186/s13007-020-00676-4 PMC7539434

[kiab188-B43] Zhang X , Henriques R, Lin SS, Niu QW, Chua NH (2006) Agrobacterium-mediated transformation of Arabidopsis thaliana using the floral dip method. Nat Protoc 1**:** 641–646 17406292 10.1038/nprot.2006.97

[kiab188-B44] Zhao J , Yu N, Ju M, Fan B, Zhang Y, Zhu E, Zhang M, Zhang K (2019) ABC transporter OsABCG18 controls the shootward transport of cytokinins and grain yield in rice. J Exp Bot 70**:** 6277–6291 31504730 10.1093/jxb/erz382 PMC6859808

[kiab188-B45] Zurcher E , Liu J, di Donato M, Geisler M, Muller B (2016) Plant development regulated by cytokinin sinks. Science 353**:** 1027–1030 27701112 10.1126/science.aaf7254

