# Supplementary material for: Phloem unloading via the apoplastic pathway is essential for shoot distribution of root-synthesized cytokinins
Source: Plant Physiol. 2021 Apr 27;186(4):2111–23. doi: 10.1093/plphys/kiab188 (PMC8331157; doi:10.1093/plphys/kiab188)
Supplement: kiab188_Supplementary_Data [file kiab188_supplementary_data.pdf]

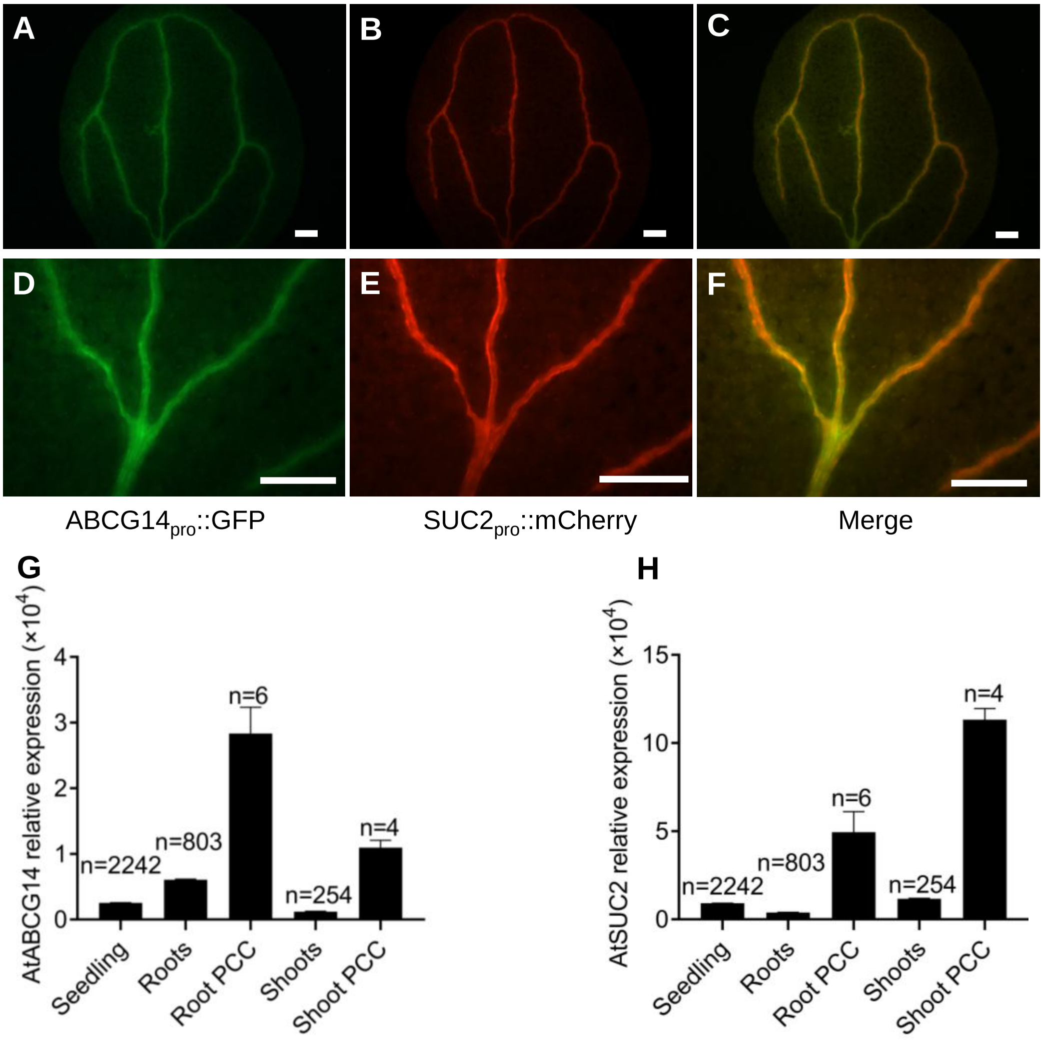

Supplemental Figure S1. *AtABCG14* and *SUC2* co-expression in the veins of *Arabidopsis* seedlings. A, GFP fluorescence. B, mCherry fluorescence. C, merged (A) and (B) signals. D, High-magnification image of (A). E, High-magnification image of (B). F, High-magnification image of (C). Scale bars = 100 µm. The seedlings for fluorescence observation *are ABCG14pro::GFP* and *SUC2pro::mCherry* double-transgenic plants. G, *AtABCG14* expression in PCC of roots and shoot; H, *AtSUC2* expression in PCC of roots and shoot. Data in (G) and (H) were extracted from Genevestigator (www.genevestigator.com). Data are presented as mean ± SE. PCC, phloem companion cells.

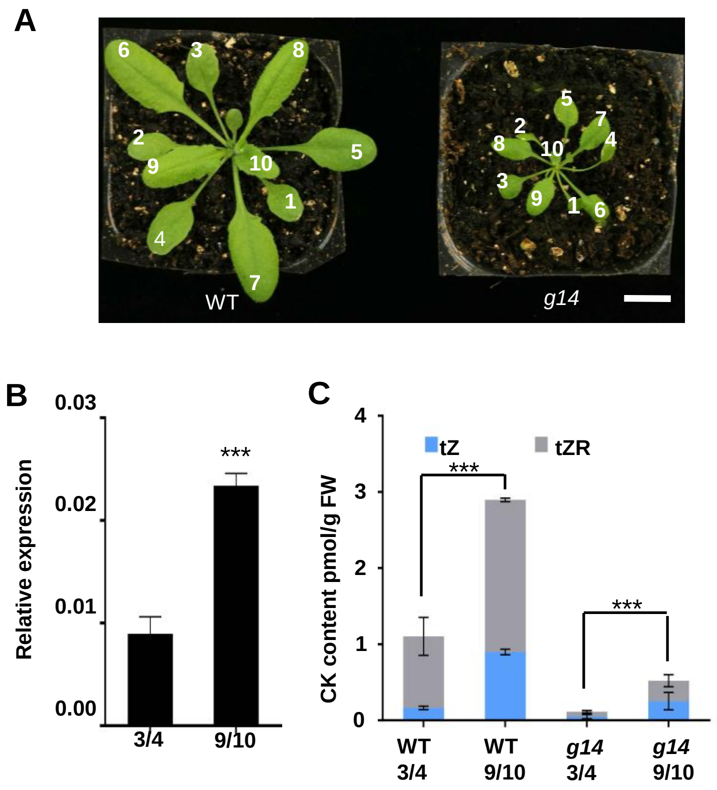

Supplemental Figure S2. Quantification of tZ and tZR in the mature and young rosette leaves of WT and *atabcg14* mutant. A, Morphological phenotypes of WT and *atabcg14* *(g14)* at 25 DAG. Oldest to youngest leaves labeled from 1 to 10. Scale bar = 1 cm. B, Quantification of *AtABCG14* expression in the $3^{rd}/4^{th}$ and $9^{th}/10^{th}$ leaves by RT-qPCR. Data are presented as mean ± SD (n = 3, biological replicates). C, tZ and tZR in mature (WT and *g14* 3/4) and growing (WT and *g14* 9/10) rosette leaves. Data are presented as mean ± SD (n = 3, biological replicates). ****P* < 0.001 (Student's *t*-test). The significant differences shown in (C) refer to the total content of tZ and tZR.

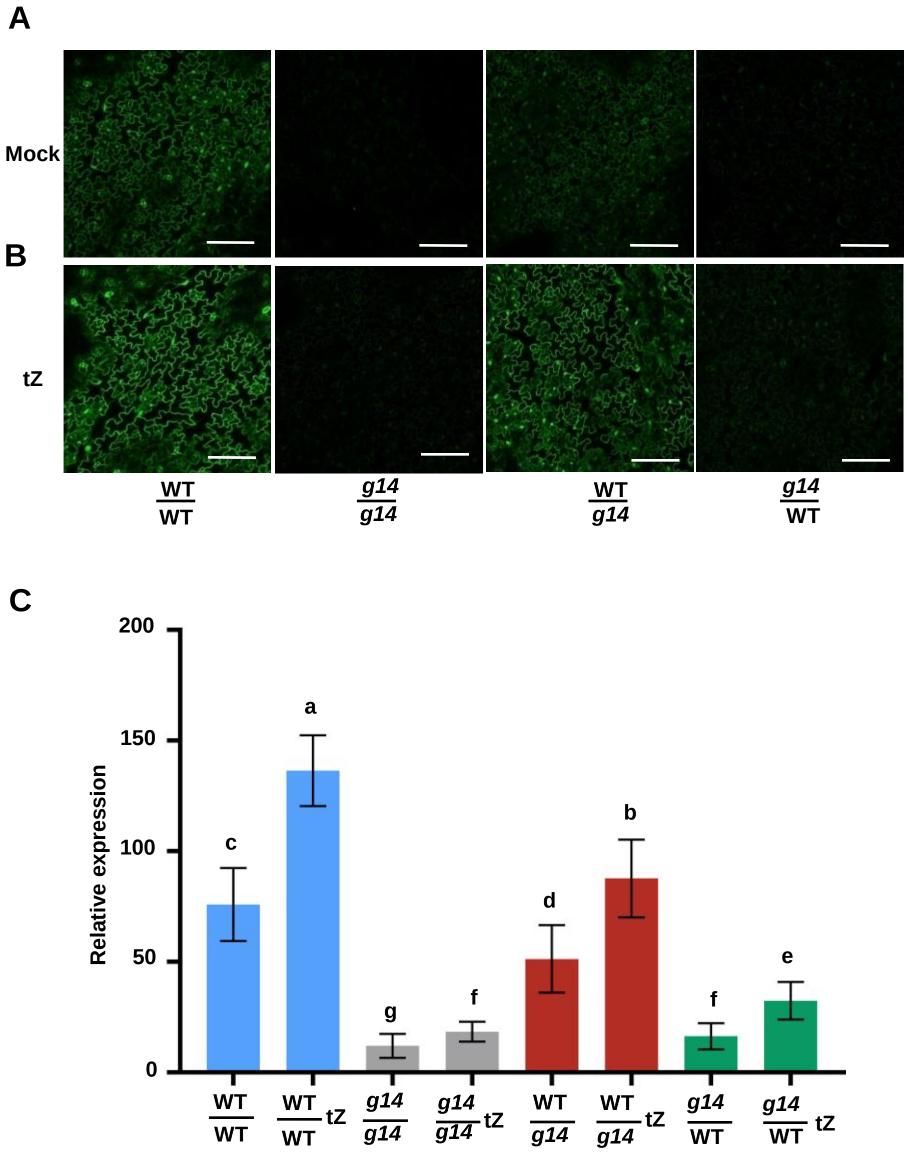

Supplemental Figure S3. GFP fluorescence of *ARR5::EGFP* transgenic plant in grafts between WT and *atabcg14* after root tZ treatment. A–B, The grafted plants treated with mock (A) and 1 μM tZ (B) for 3 h. The green fluorescence signal of each graft was obtained under same conditions. C, Quantification of GFP fluorescence in (A) using Image J software. WT/WT, self-grafted WT; WT/*g14*, WT as a scion and *atabcg14* mutant as a rootstock; *g14*/*g14*, self-grafted *atabcg14* mutant; *g14*/WT, *atabcg14* mutant as a scion and WT as a rootstock. Data are presented as mean ± SD (n = 30). The statistical analyses were performed using one-way analysis of variance (LSD test) in SPSS v. 13.0. Different letters above each column indicate significant differences ($P < 0.05$).

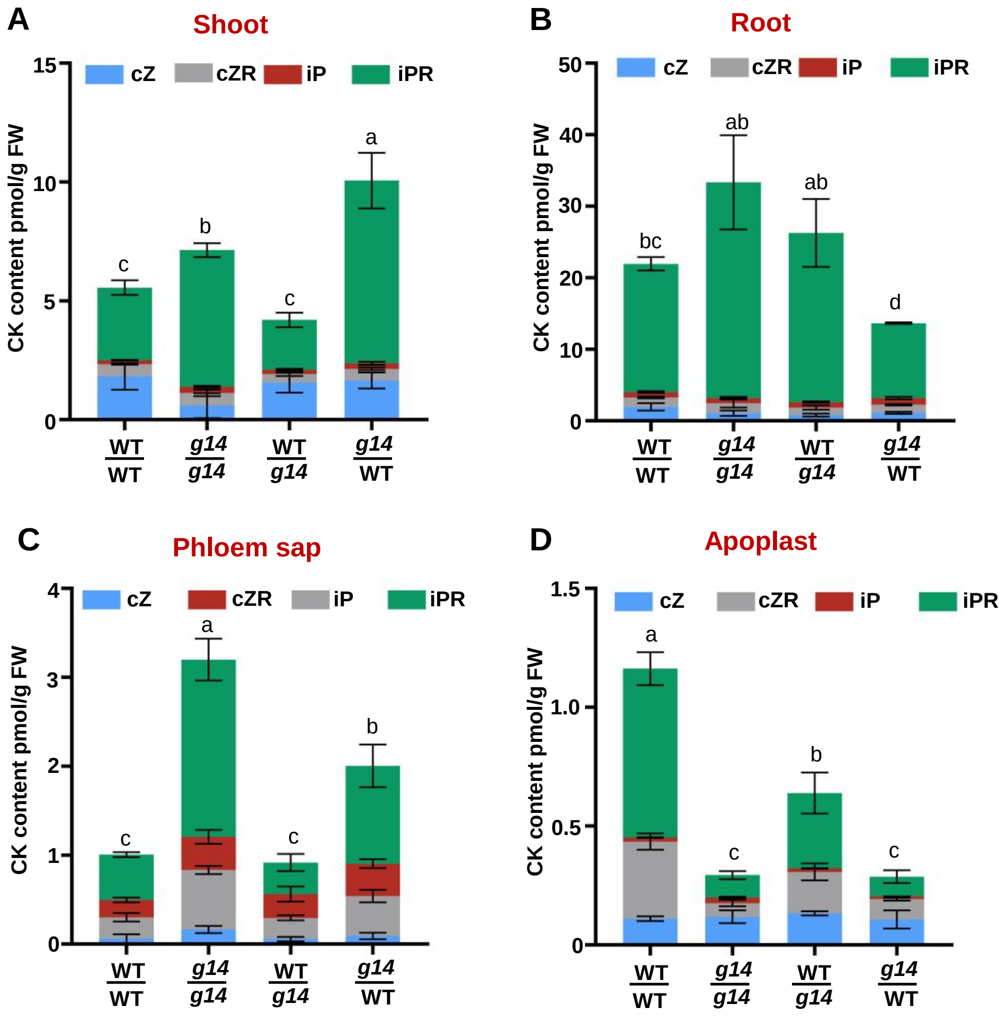

Supplemental Figure S4. Quantification of endogenous cZ, cZR, iP, and iPR in the shoots (A), roots (B), phloem saps (C), and apoplasts (D) of the grafted plants. Data are expressed as the mean ± standard deviation (SD) ($n = 3$ biological replicates; representative data of two independent experiments). Statistical analyses were performed using the one-way analysis of variance (LSD test) with SPSS software (v. 13.0). Different letters above each column indicate significant differences ($P < 0.05$). The significant differences shown in (A) to (D) refer to the total content of cZ, cZR, iP and iPR.

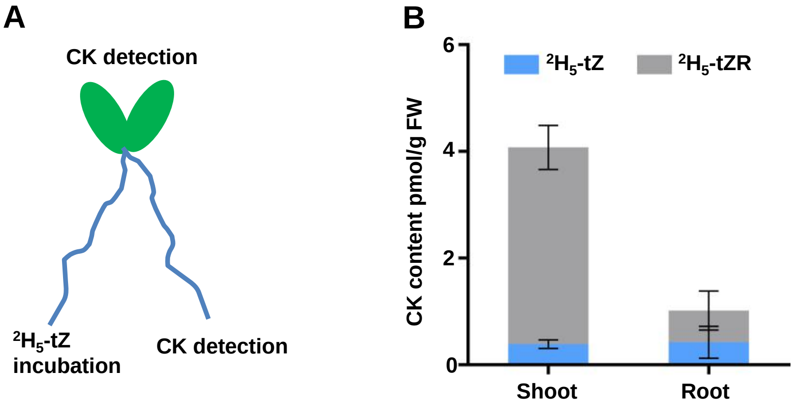

Supplemental Figure S5. Retrograde transport of tZ-type cytokinins from the shoot to root. A, The WT plants were incubated under split-root conditions with or without $^2H_5$-labeled tZ. B, Quantification of $^2H_5$-labeled tZ and tZR in the shoots and the roots (without feeding $^2H_5$-labeled tZ) after feeding $^2H_5$-tZ for 6 h in (A). Data are presented as mean $\pm$ SD (n = 3 biological replicates).

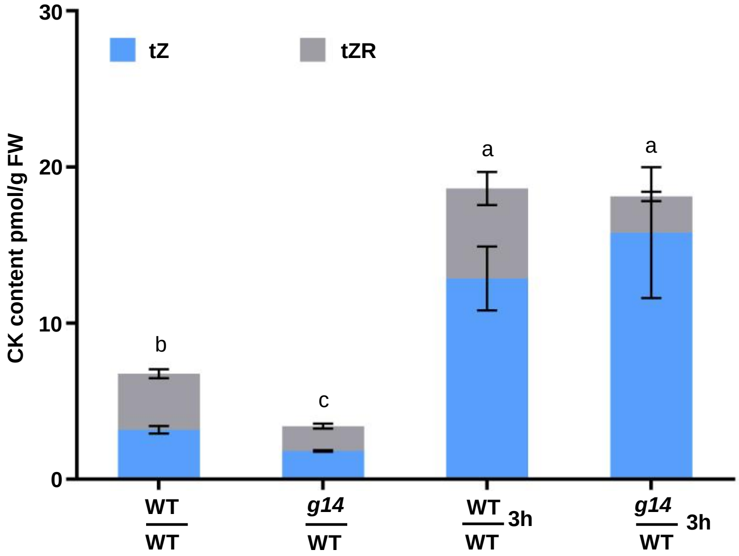

Supplemental Figure S6. Shoot cytokinin levels in the grafted plants treated with exogenous tZ in roots. Shoot cytokinin concentrations in the grafted plants treated with 10 μM tZ to the roots for 3 h. Data are presented as mean ± SD (n = 3 biological replicates). Statistical analyses were performed using the one-way analysis of variance (LSD test) with SPSS software (v. 13.0). Different letters above each column indicate significant differences ($P < 0.05$). CK, cytokinins. The significant differences shown in the figure refer to the total content of tZ and tZR.

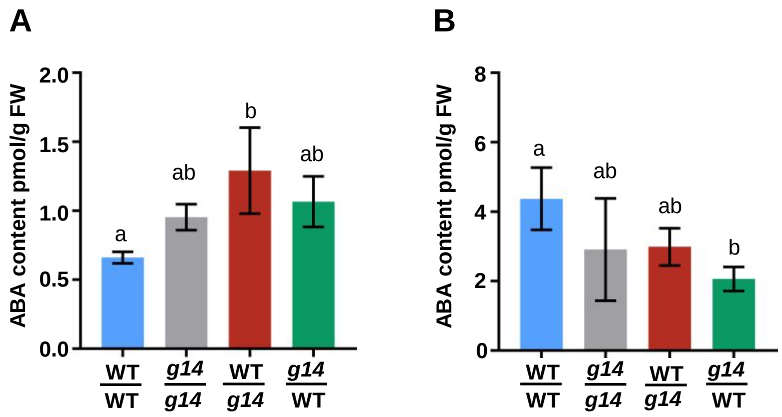

Supplemental Figure S7. Abscisic acid levels in the phloem saps and apoplastic extracts of the grafted plants. Abscisic acid concentration in the phloem saps (A) and apoplastic extracts (B) of the grafted plants. Data are presented as mean ± SD (n = 3, biological replicates, representative of two independent experiments). Statistical analyses were performed using the one-way analysis of variance (LSD test) with SPSS software (v. 13.0). Different letters above each column indicate significant differences ($P < 0.05$)

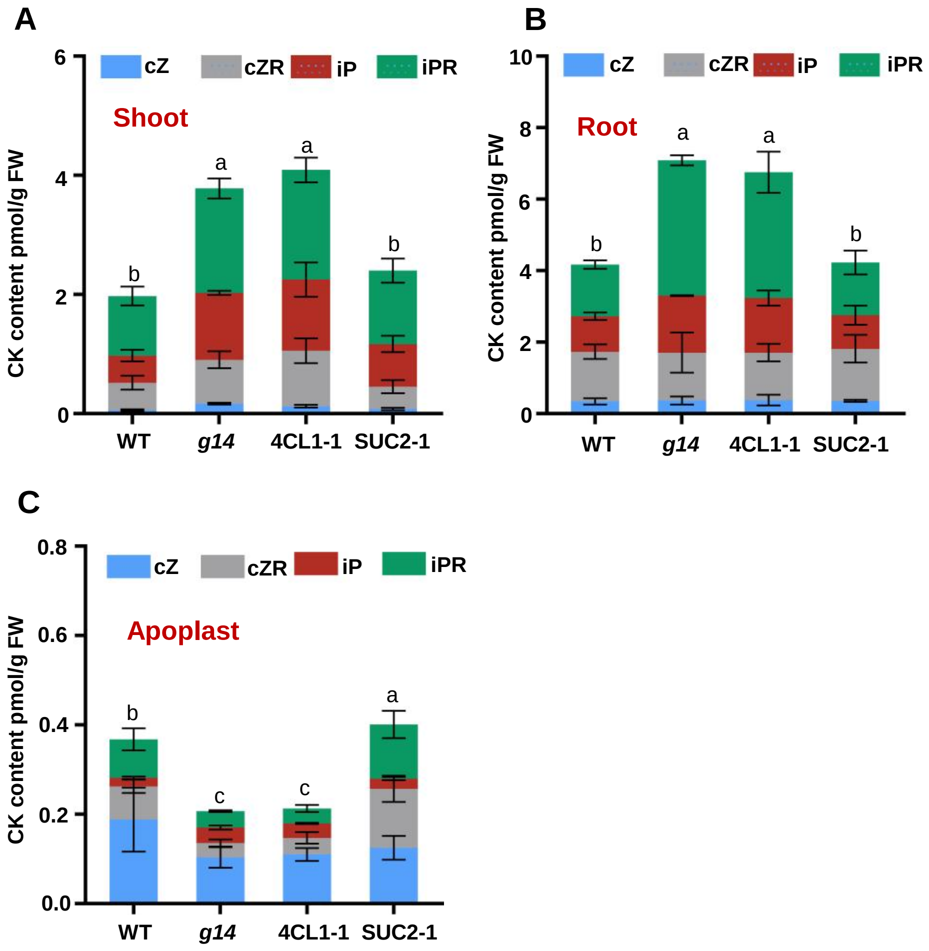

Supplemental Figure S8. Quantification of endogenous cZ, cZR, iP, and iPR in the shoots, roots and apoplast of the *atabcg14* complementary lines with *AtABCG14* under the xylem-specific promoter $4CL1_{pro}$ and phloem-specific promoter $SUC2_{pro}$. Data are presented as mean ± SD (n = 3 biological replicates; representative data from two independent experiments). Statistical analyses were performed using the one-way analysis of variance (LSD test) with SPSS software (v. 13.0). Different letters above each column indicate significant differences ($P < 0.05$). The significant differences shown in (A) to (C) refer to the total content of cZ, cZR, iP, and iPR.

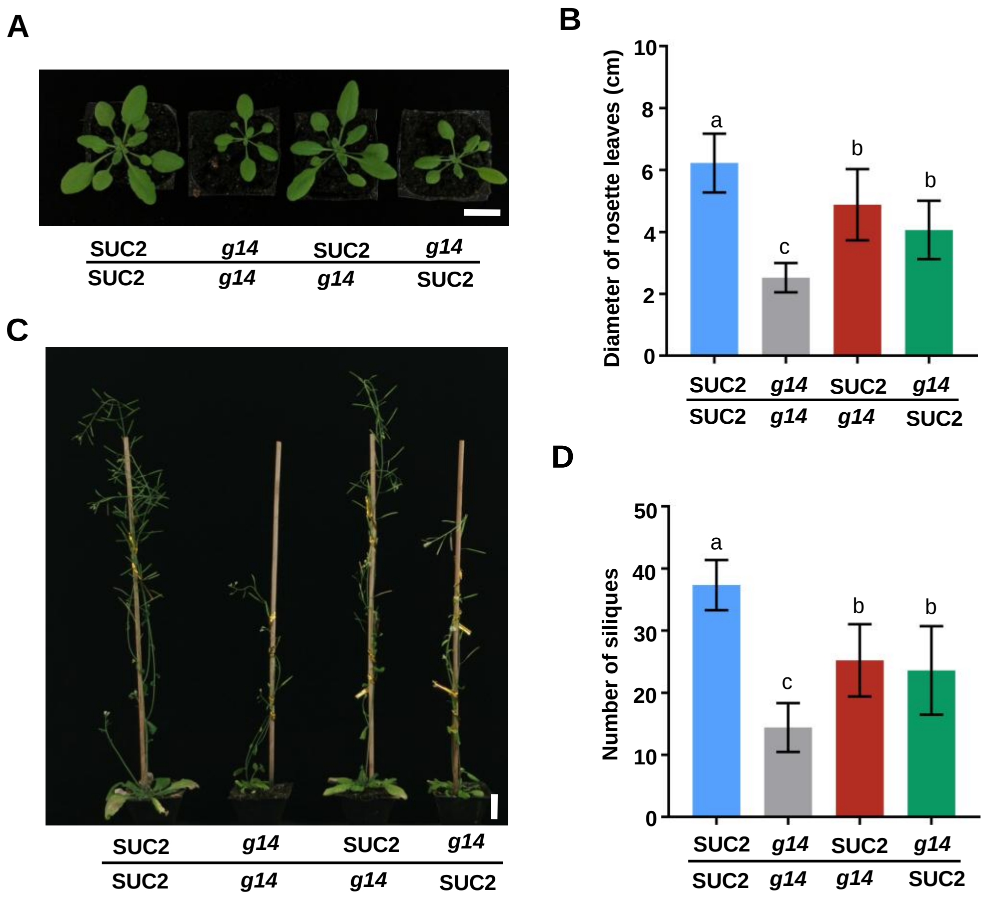

Supplemental Figure S9. *AtABCG14* expression driven by $SUC2_{pro}$ in the shoot largely rescued the growth-retarded phenotypes of *atabcg14*. A, Phenotypes of rosette leaves of grafted plants at 25 DAG. Scale bar = 2 cm. SUC2/SUC2, represents self-grafted *SUC2::AtABCG14* transgenic plants. *g14*/*g14*, represents self-grafted *atabcg14*; SUC2/*g14*, WT as scion and *atabcg14* mutant as rootstock; *g14*/*g14*, self-grafted *atabcg14* mutant; *g14*/WT, *atabcg14* mutant as scion and WT as rootstock. B, Diameter of rosette leaves of grafted plants at 25 DAG. Data are presented as mean ± SD (n = 14 independent plants); C, Phenotypes of entire grafted plants at 50 DAG. Scale bar = 2 cm. D, Silique numbers of the grafted plants at 50 DAG. Data are means ± SD (n = 15 independent plants). Statistical analyses were performed using the one-way analysis of variance (LSD test) with SPSS software (v. 13.0). Different letters above each column indicate significant differences (P < 0.05).

Supplemental Table S1 Quantification of cytokinins in leaf midrib and blade of WT/WT and *atabcg14/*WT fed $^{2}H_{5}$-tZ-type cytokinin in roots. Data are presented as mean ± SD, n = 3. *, $P < 0.05$, **, $P < 0.01$, ***, $P < 0.001$ (Student's *t*-test). WT/WT represents self-grafted WT; *g14*/WT represents graft plant with *atabcg14* as the scion and WT as the rootstock. Cytokinin concentration was calculated as pmol/g fresh weight. $^{2}H_{5}$-tZ, ^{2}H-labeled *trans*-zeatin (tZ); $^{2}H_{5}$-tZR, ^{2}H-labeled tZ riboside (tZR).

	**$^{2}H_{5}$-tZ**		**$^{2}H_{5}$-tZR**		**tZ**		**tZR**	
	**Main vein**	**Leaf blade**	**Main vein**	**Leaf blade**	**Main vein**	**Leaf blade**	**Main vein**	**Leaf blade**
**WT/WT**	0.14±0.02	0.19±0.07	0.09±0.02	0.11±0.02	0.20±0.06	0.55±0.13	0.20±0.00	0.53±0.14
***g14*/WT**	0.29±0.03**	0.14±0.01	0.06±0.00	0.05±0.01**	0.45±0.10*	0.22±0.05*	0.50±0.01***	0.17±0.02**

Supplemental Table S2 Primers used in this study.

Primer name	Primer sequences (5'-3')	Restriction site underlined	Purpose
ARR4-F	GTCGGTGGTATCGGAGGAAT		for ARR4 qRT-PCR
ARR4-R	ACGCCATCCACTATCTACCG		
ARR5-F	GTTTTGCGTCCCGAGATGTTA		for ARR5 qRT-PCR
ARR5-R	AACCGAACTATTATTCTCTCCATC		
ARR6-F	TCACCGGATCCTCTTCATGTTCTTG		for ARR6 qRT-PCR
ARR6-R	CCTCAACATCCAAACCAAGGTATTG		
ARR7-F	TTGTGGATCGTAAAGTCATCG		for ARR7 qRT-PCR
ARR7-R	CTATCAAATTCACCTTCAAATCC		
ARR15-F	GGTGGTGAAGCTGAAGAAGG		for ARR15 qRT-PCR
ARR15-R	TGGAAGATGGAGTGTCGTCA		
ARR16-F	TCAGGAGGTTCTTGTTCGTCT		for ARR16 qRT-PCR
ARR16-R	TCTGCTGTTGTCACTTTGCA		
Atactin2-F	GGTAACATTGTGCTCAGTGGTGG		Internal standard gene
Atactin2-R	CTCGGCCTTGGAGATCCACATC		
ABCG14-P1	GCTCTAGA ATGCCTCAGAACTGCATAGCAC	*Xba1*	for ABCG14 replacement of GUS in pMDC163 vector
ABCG14-P2	CGAGCTC TTACCGCAACTTCACCCGATGC	*Sac1*	
4CL1-F	CCATACTCAAAAAAGATTA		For identifying the 4CL1 promoter
4CL1-R	CATTGTAAATAGTAAATATT		
mCherry-F	GCTCTAGAATGGTGAGCAAGGGCGAGGAG	*Xba*1	for the mCherry vector
mCherry-R	CGAGCTCTTACTTGTACAGCTCGTCCAT	*Sac*1	
SUC2-F	CTGTTATAATGGACCATGAA		for identifying the SUC2 promoter
SUC2-R	ATAAATATAACTGTAAACTC		
